# Population Genomic History of the Endangered Anatolian and Cyprian Mouflons in Relation to Worldwide Wild, Feral, and Domestic Sheep Lineages

**DOI:** 10.1093/gbe/evae090

**Published:** 2024-04-27

**Authors:** Gözde Atağ, Damla Kaptan, Eren Yüncü, Kıvılcım Başak Vural, Paolo Mereu, Monica Pirastru, Mario Barbato, Giovanni Giuseppe Leoni, Merve Nur Güler, Tuğçe Er, Elifnaz Eker, Tunca Deniz Yazıcı, Muhammed Sıddık Kılıç, Nefize Ezgi Altınışık, Ecem Ayşe Çelik, Pedro Morell Miranda, Marianne Dehasque, Viviana Floridia, Anders Götherström, Cemal Can Bilgin, İnci Togan, Torsten Günther, Füsun Özer, Eleftherios Hadjisterkotis, Mehmet Somel

**Affiliations:** Department of Biological Sciences, Middle East Technical University, Ankara, Turkey; Department of Biological Sciences, Middle East Technical University, Ankara, Turkey; Department of Biological Sciences, Middle East Technical University, Ankara, Turkey; Department of Biological Sciences, Middle East Technical University, Ankara, Turkey; Department of Biochemical Sciences, University of Sassari, Sassari, Italy; Department of Biochemical Sciences, University of Sassari, Sassari, Italy; Department of Veterinary Sciences, University of Messina, Messina, Italy; Department of Biochemical Sciences, University of Sassari, Sassari, Italy; Department of Health Informatics, Graduate School of Informatics, Middle East Technical University, Ankara, Turkey; Department of Biological Sciences, Middle East Technical University, Ankara, Turkey; Department of Biological Sciences, Middle East Technical University, Ankara, Turkey; Graduate School for Evolution, Ecology and Systematics, Ludwig Maximillian University of Munich, Munich, Germany; Department of Health Informatics, Graduate School of Informatics, Middle East Technical University, Ankara, Turkey; Department of Anthropology, Hacettepe University, Ankara, Turkey; Department of Settlement Archeology, Middle East Technical University, Ankara, Turkey; Human Evolution, Department of Organismal Biology, Uppsala University, Uppsala, Sweden; Human Evolution, Department of Organismal Biology, Uppsala University, Uppsala, Sweden; Department of Veterinary Sciences, University of Messina, Messina, Italy; Department of Archaeology and Classical Studies, Stockholm University, Stockholm, Sweden; Centre for Palaeogenetics, Stockholm University, Stockholm, Sweden; Department of Biological Sciences, Middle East Technical University, Ankara, Turkey; Department of Biological Sciences, Middle East Technical University, Ankara, Turkey; Human Evolution, Department of Organismal Biology, Uppsala University, Uppsala, Sweden; Department of Anthropology, Hacettepe University, Ankara, Turkey; Agricultural Research Institute, Ministry of Agriculture, Rural Development and Environment, Nicosia, Cyprus; Department of Biological Sciences, Middle East Technical University, Ankara, Turkey

**Keywords:** sheep, genomics, conservation, domestication

## Abstract

Once widespread in their homelands, the Anatolian mouflon (*Ovis gmelini anatolica*) and the Cyprian mouflon (*Ovis gmelini ophion*) were driven to near extinction during the 20th century and are currently listed as endangered populations by the International Union for Conservation of Nature. While the exact origins of these lineages remain unclear, they have been suggested to be close relatives of domestic sheep or remnants of proto-domestic sheep. Here, we study whole genome sequences of *n* = 5 Anatolian mouflons and *n* = 10 Cyprian mouflons in terms of population history and diversity, comparing them with eight other extant sheep lineages. We find reciprocal genetic affinity between Anatolian and Cyprian mouflons and domestic sheep, higher than all other studied wild sheep genomes, including the Iranian mouflon (*O. gmelini*). Studying diversity indices, we detect a considerable load of short runs of homozygosity blocks (<2 Mb) in both Anatolian and Cyprian mouflons, reflecting small effective population size (*N*_e_). Meanwhile, *N*_e_ and mutation load estimates are lower in Cyprian compared with Anatolian mouflons, suggesting the purging of recessive deleterious variants in Cyprian sheep under a small long-term *N*_e_, possibly attributable to founder effects, island isolation, introgression from domestic lineages, or differences in their bottleneck dynamics. Expanding our analyses to worldwide wild and feral *Ovis* genomes, we observe varying viability metrics among different lineages and a limited consistency between viability metrics and International Union for Conservation of Nature conservation status. Factors such as recent inbreeding, introgression, and unique population dynamics may have contributed to the observed disparities.

**Significance**

Despite growing interest in the genetic history of sheep, two unique and isolated lineages, the Anatolian and the Cyprian mouflons, have remained mostly absent from genomic studies. Here, we present a population genomic analysis of these endangered subspecies, compared with population genomic data from other wild sheep lineages. We identify the Anatolian and Cyprian mouflon as the closest wild relatives of domestic sheep. We observe an indication of long-term low population size in the Cyprian mouflon. Finally, we find limited correlation among multiple genetic diversity metrics and with the current conservation status of the studied lineages, highlighting the difficulties of conservation-related inference from diversity data.

## Introduction

The Anthropocene has been an era of major shifts in species distributions worldwide. With the ongoing increase in human activity, excessive land use and resource exploitation are leading to an unprecedented acceleration in extinction rates. Over 40,000 species are currently considered at extinction risk (IUCN 2022), with the sixth mass extinction thought to be underway (Ceballos et al. 2015). The International Union for Conservation of Nature (IUCN) assesses current extinction risk status of species using metrics of geographic range and recent population size change estimates. Although the genetic diversity of populations and individuals is also expected to influence extinction risk, genetic information is not included in IUCN assessments, such that populations with similar IUCN metrics can differ significantly in their genetic diversity and structure (Díez-del-Molino et al. 2018; Schmidt et al. 2023). This has led to calls for closer integration of genetics with conservation assessment (Garner et al. 2020; Schmidt et al. 2023). Accordingly, maintaining and restoring genetic diversity has been included among global targets for 2030 in the recently adopted Kunming–Montreal Global Biodiversity Framework (GBF) (United Nations Environment Programme 2022).

A major phenomenon that conservation genetics centers on is the process of genome erosion with population decline. Small and fragmented populations become more prone to the detrimental effects of genetic drift and inbreeding, leading to A and F type extinction vortices, which are characterized by an increase in a species' deleterious mutation load and a decrease in the adaptive potential, respectively (Frankham 2005; Nabutanyi and Wittmann 2021). These extinction-related dynamics can be detected as reduction in heterozygosity, elevated ratios of *P*_n_/*P*_s_ (number of nonsynonymous polymorphisms/number of synonymous polymorphisms), and long runs of homozygosity (ROH). However, factors such as different demographic histories or generation intervals can lead to differing levels and patterns of genome erosion signals among declining taxa (Bosse and van Loon 2022). For instance, depending on the nature of population decline, such as sudden bottlenecks, repeated founder effects, or long-term small effective population size (*N*_e_), one may or may not observe a deflated *P*_n_/*P*_s_ ratio; this is because the decline in the force of purifying selection caused by bottlenecks and the purging of recessive deleterious genetic load under sustained small *N*_e_ can have opposing effects (Bertorelle et al. 2022). Therefore, assessing population viability using genetic data is not always straightforward and requires joint analysis of multiple parameters.

Asiatic (ASM) and European (EUM) mouflons, i.e. wild-living close relatives of domestic sheep (DOM), represent an interesting case for studying conservation genetics, with closely related lineages with distinct histories and conservation status. The ASM (*Ovis gmelini*; formerly *Ovis orientalis*) is currently represented by three to five subspecies ranging through Armenia, Iraq, Iran, Turkey, and Cyprus (Garel et al. 2022). This group is considered to include the closest living relatives of the wild source population of DOM (Hiendleder et al. 2002; Chessa et al. 2009; Cheng et al. 2023; Wang et al. 2023). The ASM is listed as “Near Threatened” under the IUCN Red List criteria as of 2024, with most populations numbering in the thousands.

The Cyprian mouflon (CYM) (*Ovis gmelini ophion*) and Anatolian mouflon (ANM) (*Ovis gmelini anatolica*) are two endemic subspecies of the ASM. They are considered both genetically and phenotypically closely related to each other (Hadjisterkotis et al. 2016). Both have undergone bottlenecks over the last few decades, rendering them vulnerable to the detrimental effects of genetic drift and inbreeding (Hadjisterkotis and Bider 1993; Arıhan and Bilgin 2001; Hadjisterkotis 2001; Özdirek 2009; Özüt 2009; Michel and Ghoddousi 2020). Both *O. g. ophion* and *O. g. anatolica* are considered “Endangered” by the IUCN due to their small population sizes and continuing decline (Michel and Ghoddousi 2020). The CYM is also listed in the Convention on International Trade in Endangered Species of Wild Fauna and Flora (CITES) appendix (Michel and Ghoddousi 2020).

The mouflon term is also used for the EUM (*Ovis gmelini musimon*). Although the native EUM populations were found in Sardinia and Corsica, these wild-living sheep are found today across Europe through recent human introduction. The EUM is generally recognized to represent feral populations of the earliest DOM stocks brought to Europe by people in the Early Neolithic (see Garel et al. 2022 for a full discussion). Indeed, the group clusters with DOM *Ovis aries* based on their mitogenome (Townsend et al. 2019). The EUM has not been assessed by the IUCN due to its assumed feral status; still, there are local conservation efforts for the two primary natural populations in Corsica and Sardinia (Mereu et al. 2019; Satta et al. 2021; Barbato et al. 2022; Portanier et al. 2022).

Our study focuses on the history and population origins of the ANM and CYM, questions which are little understood. Zooarchaeological records of sheep in Southwest Asia point to Central/Eastern Anatolia as putative regions of domestication during the Early Neolithic (∼10,000 BP) (Zeder 2008; Abell et al. 2019); the ANM may be a sister clade to DOM (source of the original domestic stock) or a feral relic of these ancient domesticates (Demirci et al. 2013; Her et al. 2022). Meanwhile, the earliest zooarcheological record of sheep in Cyprus already dates back to ∼10,000 BP (Zeder 2008) (supplementary note 1, Supplementary Material online). These sheep possibly originate from Anatolia or the Levant; they are thought to have been brought at the early stages of sheep domestication and later became feral (Vigne et al. 2011, 2014; Guerrini et al. 2015; Sanna et al. 2015).

Once widespread in their homelands, both ANM and CYM were driven to near extinction in the 20th century due to excessive hunting, poaching, habitat loss, niche overlaps with DOM/goat, and predation by stray dogs (Hadjisterkotis and Bider 1993) (supplementary note 1, Supplementary Material online). Both populations experienced intense bottlenecks, with the number of CYM individuals reduced to ∼40 in the 1930s and those of ANM down to ∼50 in the 1960s (Turan 1984; Hadjisterkotis 1992, 2001; Kence and Tarhan 1997; Arıhan 2000; Nicolaou et al. 2016; Michel and Ghoddousi 2020). Through conservation efforts, population sizes have later rebounded and are today estimated to be 2,500 to 3,000 for CYM and ∼1,200 for ANM (Michel and Ghoddousi 2020; Kapnisis et al. 2022) (Çelik M, personal communication). The ANM is currently found at eight small reserves, seven of which are reintroduction sites, while the CYM is confined to only one reserve (Hadjisterkotis 1992, 2001) (Çelik M, Hatipoğlu T, Emir H, personal communication). Both subspecies are legally protected, but seasonally regulated hunting has been permitted in Turkey in the recent past.

Here, we study the population history and diversity in the endemic ANM and CYM populations using published and newly generated genomic data. Joining this data with data from eight other extant sheep lineages, we investigate divergence and gene flow, as well as various metrics of diversity, population size estimates, and mutation load. We specifically ask whether the genetic diversity landscapes of Anatolian and Cyprian sheep are shaped by differences in mainland versus island dynamics (e.g. isolation as well as the absence of natural predators and competition) or whether recent severe bottlenecks and genome erosion may have created similar diversity landscapes. In addition to this question, we further compare these metrics and IUCN status among the studied sheep lineages.

## Results

**Fig. 1.**
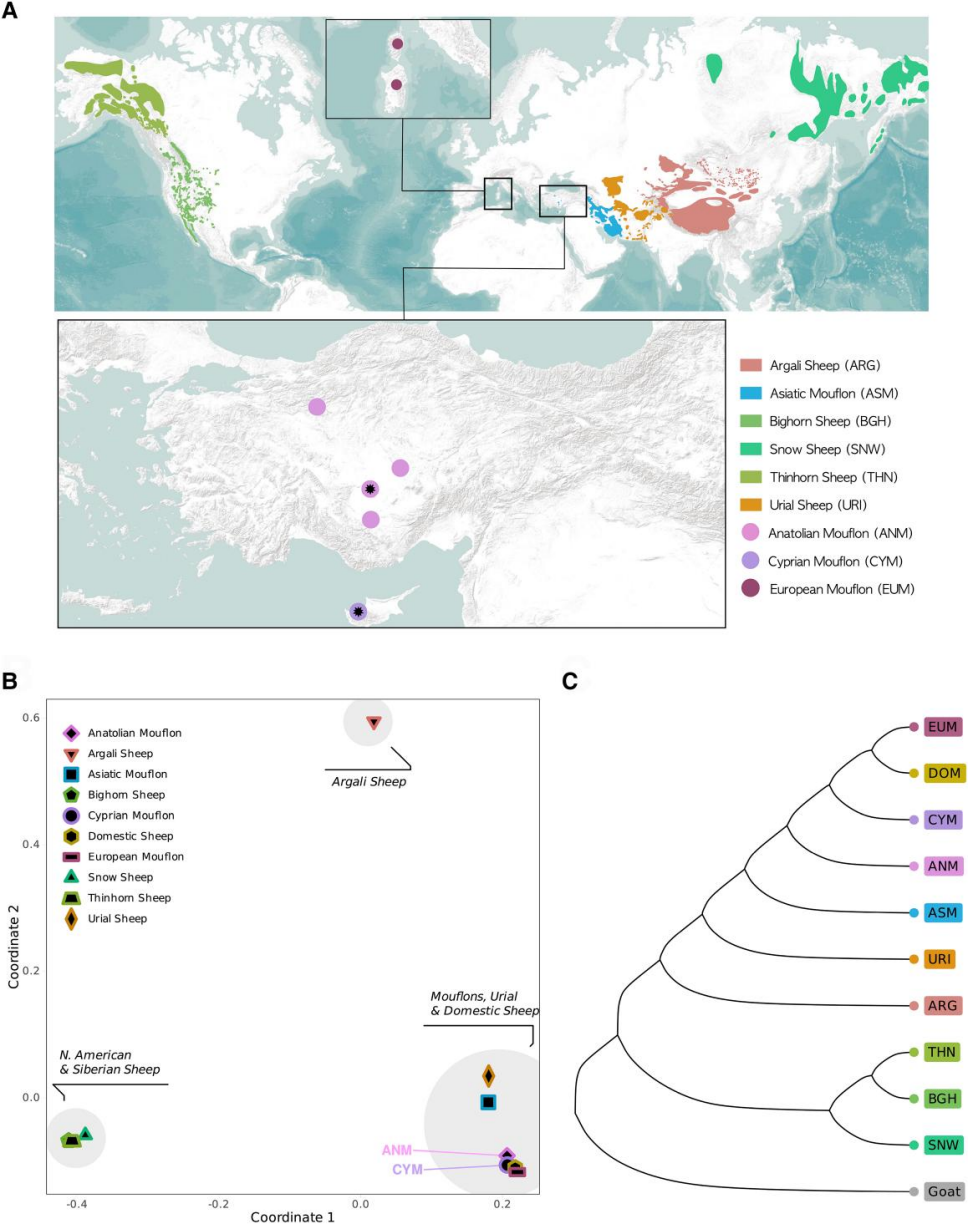
The geographic distribution and phylogeny of the studied sheep lineages. A) Geographic distribution of the sheep species considered wild by IUCN (data from IUCN). The distribution ranges of the CYM and ANM are shown separately as points. The two points with the additional symbols in the middle denote the sampling locations. The primary populations of the feral-considered EUM are also shown separately as points in the smaller panel. B) MDS analysis of the studied sheep lineages, using 1-outgroup-f3 statistics as distance proxy. C) NJ tree of the studied sheep lineages, using (1-outgroup-f3) as distance proxies and using goat as outgroup. Bootstrap support was calculated using 500 replicates and all branches have 100% support.

We generated whole genome data for *n* = 8 CYM samples with a median coverage of 2.6× (ranging from 1.8× to 17.4×). We combined this with publicly available genomes from *n* = 2 CYM, *n* = 5 ANM, *n* = 6 ASM from Iran, *n* = 3 EUM, *n* = 6 argali sheep (ARG), *n* = 6 bighorn sheep (BGH), *n* = 6 snow sheep (SNW), *n* = 5 thinhorn sheep (THN), *n* = 4 urial sheep (URI), and *n* = 6 DOM individuals. Our data set thus included *n* = 57 sheep genomes in total (Fig. 1A, Table 1) (Kijas et al. 2012; Deng et al. 2020; Li et al. 2020; Chen et al. 2021). The six domestic breeds were from Asia, the Middle East, Europe, and Africa, chosen to reflect the global diversity of this group. Sequence reads were mapped to the DOM reference genome Oar_v4.0, genome-wide coverages ranging between 0.4× to 17.4× (median = 7.5×) (Table 1).

**Table 1:** General information on the newly generated and published genomes

Sample ID	Lineage	Taxonomy	Region	Coverage	Sex	Source	Project IDs
cym002	CYM	*O. g. ophion*	Cyprus	2,616	XY	This study	PRJEB69690
cym003	CYM	*O. g. ophion*	Cyprus	3,203	XY	This study	PRJEB69690
cym004	CYM	*O. g. ophion*	Cyprus	4,143	XY	This study	PRJEB69690
cym006	CYM	*O. g. ophion*	Cyprus	2,629	XX	This study	PRJEB69690
cym007	CYM	*O. g. ophion*	Cyprus	2,559	XY	This study	PRJEB69690
cym008	CYM	*O. g. ophion*	Cyprus	17,435	XY	This study	PRJEB69690
cym009	CYM	*O. g. ophion*	Cyprus	2,547	XX	This study	PRJEB69690
cym011	CYM	*O. g. ophion*	Cyprus	2,915	XX	This study	PRJEB69690
cym012	CYM	*O. g. ophion*	Cyprus	2,702	XX	Kaptan D, Atağ G, Vural KB, Morell Miranda P, Akbaba A, et al., in preparation	PRJEB69690
cym013	CYM	*O. g. ophion*	Cyprus	1,801	XY	Kaptan D, Atağ G, Vural KB, Morell Miranda P, Akbaba A, et al., in preparation	PRJEB69690
OGA009	ANM	*O. g. anatolica*	Turkey	0.691	XX	Kaptan D, Atağ G, Vural KB, Morell Miranda P, Akbaba A, et al., in preparation	PRJEB69690
OGA014	ANM	*O. g. anatolica*	Turkey	1,020	XX	Kaptan D, Atağ G, Vural KB, Morell Miranda P, Akbaba A, et al., in preparation	PRJEB69690
oga018	ANM	*O. g. anatolica*	Turkey	15,598	XX	Kaptan D, Atağ G, Vural KB, Morell Miranda P, Akbaba A, et al., in preparation	PRJEB69690
OGA021	ANM	*O. g. anatolica*	Turkey	0.555	XX	Kaptan D, Atağ G, Vural KB, Morell Miranda P, Akbaba A, et al., in preparation	PRJEB69690
OGA022	ANM	*O. g. anatolica*	Turkey	0.404	XX	Kaptan D, Atağ G, Vural KB, Morell Miranda P, Akbaba A, et al., in preparation	PRJEB69690
				…			
YZ.11	ASM	*O. gmelini*	Iran	14,823	XX	Li et al. 2020	PRJNA624020
YZ.12	ASM	*O. gmelini*	Iran	11,912	XX	Li et al. 2020	PRJNA624020
TH.1	ASM	*O. gmelini*	Iran	14,618	XY	Li et al. 2020	PRJNA624020
KR.6	ASM	*O. gmelini*	Iran	13,485	XY	Li et al. 2020	PRJNA624020
266	ASM	*O. gmelini*	Iran	12,370	XY	Li et al. 2020	PRJNA624020
SH-7	ASM	*O. gmelini*	Iran	11,101	XY	Li et al. 2020	PRJNA624020
MUF1	EUM	*O. a. musimon*	Finland	7,986	XX	Chen et al. 2021	PRJNA764308
MUF2-1	EUM	*O. a. musimon*	Finland	8,374	XX	Chen et al. 2021	PRJNA764308
MUF3-1	EUM	*O. a. musimon*	Finland	7,319	XX	Chen et al. 2021	PRJNA764308
ARG20	ARG	*Ovis ammon*	China	7,396	XY	Deng et al. 2020	PRJNA645671
ARG3-1	ARG	*O. ammon*	China	7,925	XY	Deng et al. 2020	PRJNA645671
ARG23	ARG	*O. ammon*	China	7,544	XY	Deng et al. 2020	PRJNA645671
ARG8-2	ARG	*O. ammon*	China	6,731	XY	Deng et al. 2020	PRJNA645671
ARG9-3	ARG	*O. ammon*	China	7,896	XY	Deng et al. 2020	PRJNA645671
KS1	ARG	*Ovis ammon polii*	China	15,730	XY	Yang et al. 2017	PRJNA391748
bighorn1	BGH	*Ovis canadensis*	Canada	7,310	XY	Chen et al. 2021	PRJNA764308
bighorn2	BGH	*O. canadensis*	Canada	8,158	XX	Chen et al. 2021	PRJNA764308
bighorn3	BGH	*O. canadensis*	Canada	8,132	XY	Chen et al. 2021	PRJNA764308
bighorn4	BGH	*O. canadensis*	Canada	8,132	XX	Chen et al. 2021	PRJNA764308
bighorn5	BGH	*O. canadensis*	Canada	7,878	XY	Chen et al. 2021	PRJNA764308
Ovican1 (BS48)	BGH	*O. canadensis*	…	8,303	XY	Broad Institute	PRJNA399410
snow7	SNW	*Ovis nivicola*	Siberia	6,834	XX	Chen et al. 2021	PRJNA764308
snw1	SNW	*O. nivicola*	Siberia	6,969	XX	Chen et al. 2021	PRJNA764308
snow13	SNW	*O. nivicola*	Siberia	6,741	XY	Chen et al. 2021	PRJNA764308
snow5	SNW	*O. nivicola*	Siberia	7,746	XY	Chen et al. 2021	PRJNA764308
snow6	SNW	*O. nivicola*	Siberia	7,218	XX	Chen et al. 2021	PRJNA764308
snow8	SNW	*O. nivicola*	Siberia	8,084	XY	Chen et al. 2021	PRJNA764308
Thinhorn1	THN	*Ovis dalli*	Canada	8,521	XY	Chen et al. 2021	PRJNA764308
Thinhorn3	THN	*O. dalli*	Canada	9,572	XY	Chen et al. 2021	PRJNA764308
Thinhorn4	THN	*O. dalli*	Canada	8,425	XY	Chen et al. 2021	PRJNA764308
Thinhorn5	THN	*O. dalli*	Canada	8,100	XY	Chen et al. 2021	PRJNA764308
Thinhorn9	THN	*O. dalli*	Canada	7,519	XY	Chen et al. 2021	PRJNA764308
BJ3-1	URI	*Ovis vignei*	Iran	6,870	XX	Chen et al. 2021	PRJNA764308
BJ4-1	URI	*O. vignei*	Iran	7,524	XX	Chen et al. 2021	PRJNA764308
BJR5	URI	*O. vignei*	Iran	6,901	XX	Chen et al. 2021	PRJNA764308
BJ1-2	URI	*O. vignei*	Iran	6,826	XX	Chen et al. 2021	PRJNA764308
AL118	Altay sheep	*O. aries*	China	11,724	XX	Li et al. 2020	PRJNA624020
FINN305	Finnsheep	*O. aries*	Finland	12,718	XX	Li et al. 2020	PRJNA624020
Sc12	Mbororo sheep	*O. aries*	Cameroon	16,662	XY	Li et al. 2020	PRJNA624020
DLS249	Duolang	*O. aries*	China	11,285	XX	Li et al. 2020	PRJNA624020
CC50	Cine-Capari	*O. aries*	Turkey	6,432	XY	Kijas et al. 2012	PRJNA160933
AWA-33	Awassi	*O. aries*	Iraq	7,192	XY	Deng et al. 2020	PRJNA645671
BAT_IOSW	Goat	*Capra hircus*	Morocco	5,013	XY	NextGen project	PRJEB3134

The project IDs refer to the ENA.

Genetic sex was determined with the *R_x_* metric (Mittnik et al. 2016), which compares autosomal versus X chromosome (chrX) coverages (supplementary table S2, Supplementary Material online). However, we could not use *R_x_* thresholds for sex identification chosen for humans, most likely due to the relatively incomplete nature of the sheep genome assembly; we therefore optimized these to suit the sheep data (Materials and Methods). All five ANM and four of the ten CYM individuals were female. We also estimated relatedness among individuals using the program *READ* (Kuhn et al. 2018) and found one pair of genetically identical genomes (possible twins or sample duplicates) among the CYM individuals (supplementary table S3, Supplementary Material online). One individual from this pair was excluded from the analyses to ensure the independence of the sample. We also found one pair of possible second-degree and eight pairs of possible third-degree relative CYM pairs, among which we excluded two individuals (Materials and Methods).

To study genetic variation, we created a single nucleotide polymorphism (SNP) data set representing all the sheep populations while minimizing biases due to heterogeneity in sample size and coverage among lineages. For this, we called SNPs using one representative genome from each of the ten lineages with similar data quality downsampled to 7.5 to 8.5× (Table 1, supplementary table S1, Supplementary Material online) and performed de novo SNP calling on each of these ten, resulting in ∼15 million (15 M) SNPs after filtering (Materials and Methods). We then genotyped the remaining individuals at these positions, combined the data, and applied further filtering to obtain a sheep variation data set comprising ∼14 million autosomal SNPs (Materials and Methods). The data set was used for calculating *f*-statistics, calling ROH segments and determining biologically related individuals. We note that this approach limits ascertainment bias compared with calling SNPs from the full data set but does not fully eliminate such bias as the lineages are not equally distant from each other. In addition, we validated our main findings possibly prone to ascertainment bias using a ∼116k subset of the ∼14 M SNPs that were identified as heterozygous in a goat individual (we lacked an outgroup phylogenetically close enough for large-scale SNP ascertainment) (Materials and Methods).

### Population Affinities

We studied demography using *f_3_* and *f_4_* statistics, which measure the amount of shared drift among tested populations (Patterson et al. 2012). To summarize genetic differentiation between pairs of populations, we calculated genetic distances using 1-outgroup-*f_3_* statistics of the form *f_3_*(Goat; Pop1, Pop2), where Pop1 and Pop2 are any populations among the studied sheep lineages (supplementary table S3, Supplementary Material online). We found that ANM and CYM were most distant to sheep lineages from North America and Siberia and showed the highest affinity to EUM and DOM. We also observed that *f_3_*(Goat; Pop1, CYM) and *f_3_*(Goat; Pop1, ANM) values, where Pop1 is any other sheep lineage, were highly correlated (supplementary fig. S3 and table S4, Supplementary Material online). Employing multidimensional scaling (MDS) to summarize these outgroup-*f_3_*-based distances, we observed three separate clusters (Fig. 1B, supplementary fig. S1, Supplementary Material online). North American and Siberian wild sheep comprised one cluster and the ARG another, positioned separately from all other sheep. In the third cluster, CYM and ANM grouped with the other mouflons, as well as with URI and DOM. We also utilized 1-outgroup-*f_3_* values to construct a neighbor joining (NJ) tree (all branches had 100% support) (Fig. 1C). In this tree, CYM formed a clade with the EUM and DOM, while the ANM was a sister lineage to this clade. We validated these clustering patterns in the MDS and the NJ tree with the data set of goat heterozygous SNPs (supplementary fig. S2, Supplementary Material online). We further confirmed these patterns by calculating *f_4_* statistics of the form *f_4_*(Goat, DOM/EUM; ANM, CYM) and *f_4_*(Goat, CYM; ANM, DOM/EUM) (supplementary fig. S4 and tables S5 and S6, Supplementary Material online). These results overall demonstrate that DOM and EUM (which are considered feral populations of past domestic livestock) show higher affinity to the CYM than ANM, and reciprocally, the CYM is closer to DOM and EUM than to ANM. Meanwhile, both ANM and CYM appeared to share a similar amount of drift with other wild sheep lineages.

We also analyzed the mitochondrial DNA (mtDNA) similarity patterns by using a median-joining (MJ) network. This revealed three distinct branches (supplementary fig. S5, Supplementary Material online). BGH, SNW, and THN were clustered on one branch; meanwhile, ARG, URI, and three ASM individuals clustered on another branch. The last branch was composed of DOM, CYM, ANM, and EUM and the remaining three ASM individuals. Within this third clade, DOM were clustered into five different haplogroups (hpg) named A to E (Meadows et al. 2007). HpgB is the most widely observed hpg among European DOM and hpgA among DOM from Asia in general, while hpgC is relatively frequent among DOM from the Caspian Sea, the Middle East, and northern China (Lv et al. 2015). EUM samples were clustered with hpgB. Meanwhile, ANM clustered in two different subbranches, *n* = 3 individuals (OGA009, oga018, OGA022) were on hpgA, and *n* = 2 individuals (OGA014, OGA021) were on a distinct branch near hpgC and hpgE, named hpgX (Demirci et al. 2013). Finally, all CYM samples were clustered near hpgX. Notably, these mtDNA patterns differ from the autosomal clustering in the sense that CYM clusters with ANM rather than with DOM/EUM, suggesting different population histories on the maternal line.

### Diversity

**Fig. 2.**
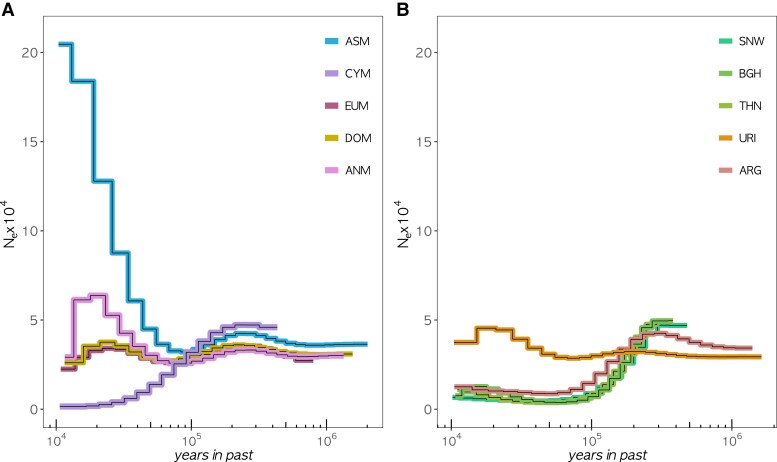
Population size changes among sheep lineages. PSMC analysis of high-coverage individuals from each lineage, A) for mouflons and domestic sheep and B) for N. American, Siberian, and Asian wild sheep. The *x* axis shows time in a log scale, and the *y* axis shows the estimated effective population size. We assumed a generation time of 3 years and a mutation rate of 1.5 × 10^−8^.

The ANM and CYM populations are thought to have undergone severe population declines in the recent past, which should leave detectable signatures in their genomic diversity data. Employing the pairwise sequential Markovian coalescent (PSMC) approach to the highest coverage individuals (cym008 = 17.4× and oga018 = 15.6×), we estimated the change in past effective population sizes (*N*_e_). The demographic trajectory of the CYM showed a monotonic decline in *N*_e_ followed by stabilization near 10 kya (thousand years ago) (Fig. 2). Meanwhile, ANM shows a period of population growth starting from 50 kya, followed by a sudden decline near 10 kya. Considering the sensitivity of the PSMC approach to genome-wide coverage (Nadachowska-Brzyska et al. 2016), we also ran a trial with samples downsampled to similar coverages (7.5 to 8.5×). Although the trajectories inferred from the original and downsampled data were partly different, the main qualitative patterns were similar: we still observed a long period of *N*_e_ decline in CYM and a period of population growth in ANM (supplementary fig. S6, Supplementary Material online). In both results, compared with the most recent *N*_e_ values estimated from individuals of DOM and other wild sheep populations, CYM shows the lowest estimate. We note, however, that PSMC results should not be taken at face value as they can be influenced by technical factors as well as admixture (Li and Durbin 2011).

**Fig. 3.**
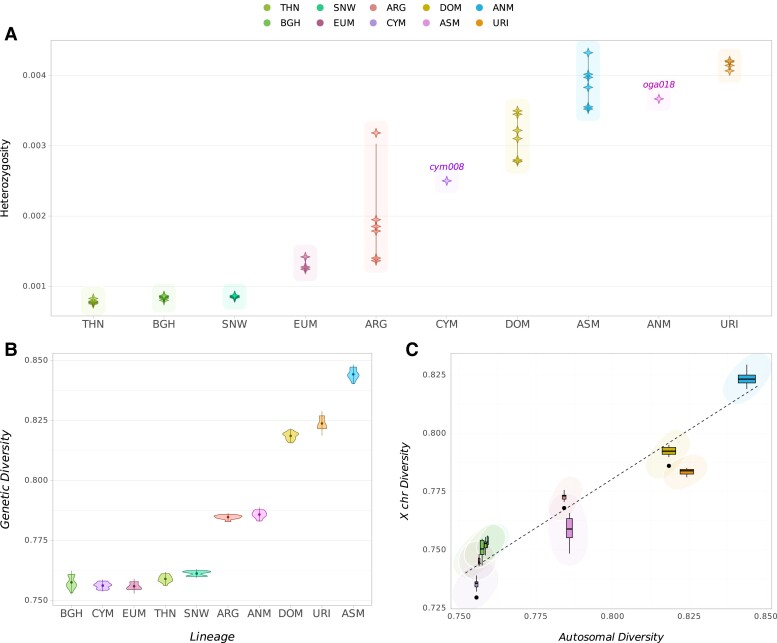
Heterozygosity and diversity estimates among sheep lineages. A) Genome-wide heterozygosity values estimated using genotype likelihoods. Only the high-coverage genomes cym008 and oga018 were included to represent CYM and ANM, respectively. B) Within-population autosomal diversity values estimated using pairwise 1-outgroup-f3 statistics per lineage. C) Comparison of autosomal versus chrX diversities, each estimated using pairwise 1-outgroup-f3 statistics. The regression line was generated with the loess algorithm in the R stats package.

Next, we studied within-population genetic diversity patterns in CYM and ANM and compared these with other sheep lineages. For this, we used both genome-wide heterozygosity (π) and interindividual diversity estimates using pairwise 1-outgroup-*f_3_* statistics (Fig. 3). For π, we used the highest coverage individuals (cym008 and oga018) for CYM and ANM and ran the analyses on genomes downsampled to similar coverages (6.5 to 7.5×). The North American/Siberian group had the lowest π followed by the EUM (Fig. 3A, supplementary table S7, Supplementary Material online). The CYM and ANM recorded moderate and high estimates, respectively. The highest value was observed for the URI, more than four times higher than those of North American/Siberian lineages.

In order to include the low-coverage CYM and ANM individuals in the analyses, we then estimated interindividual diversities using pairwise 1-outgroup-*f_3_* statistics between individuals within each population, a proxy for population-wide heterozygosity (Fig. 3B). The highest interindividual diversity was observed for the ASM from Iran, which is thought to have experienced gene flow from other wild and domestic sheep populations (Chen et al. 2021; Morell Miranda et al. 2023). The CYM showed one of the lowest diversity estimates along with the EUM and the North American/Siberian group. The ANM had relatively moderate level of interindividual diversity. We observed qualitatively similar results using the data set of goat heterozygous SNPs (supplementary fig. S2, Supplementary Material online).

We further studied mtDNA and chrX diversities across these genomes, by calculating average pairwise differences (π) on mtDNA and pairwise 1-outgroup-*f_3_* statistics with the chrX data set (supplementary figs. S7 to S9, Supplementary Material online). These showed similar patterns to autosomal diversity estimates, with CYM showing the lowest and ANM moderate values compared with other sheep.

We then compared chrX diversities with autosomal diversities across the ten lineages (Fig. 3C, supplementary fig. S8, Supplementary Material online). The autosome/chrX ratios ranged between 1.01 and 1.05, lower than the expected proportion of 1.33 assuming equal *N*_e_ for both sexes (supplementary fig. S8, Supplementary Material online). These values suggest smaller male *N*_e_, consistent with the polygynous mating structure in sheep (Corlatti and Lovari 2023). However, the lineages also varied among themselves: we found that the autosomal/chrX diversity ratios were closest to 1 in the N. American/Siberian group, followed by the EUM and ARG. This may be compatible with a relatively higher female contribution to the genetic variation in these populations, i.e. a smaller relative male *N*_e_. In contrast, other populations including CYM and ANM showed relatively higher autosomal/chrX diversity ratios, implying that male versus female *N*_e_ differences may be more modest in these groups relative to sheep from N. America/Siberia.

### Inbreeding

**Fig. 4.**
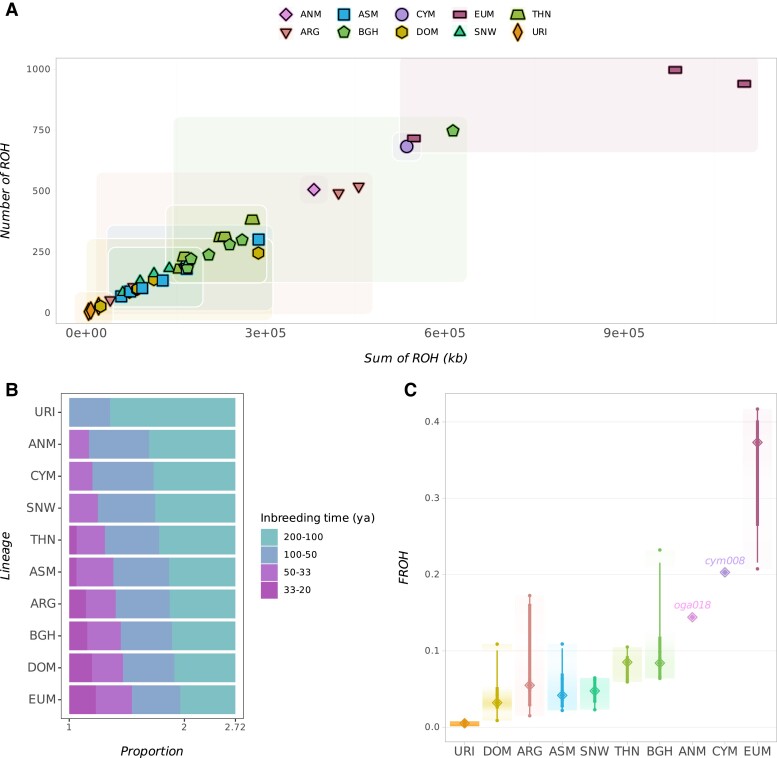
ROH in sheep genomes. A) Number of ROH segments >500 kb plotted against the total length of the segments found in each individual. The Anatolian and Cyprian populations are only represented by the high-coverage individuals oga018 and cym008, respectively. B) Size distribution of ROH segments divided into four classes (0.5 to 1, 1 to 2, 2 to 3, and 3 to 5 Mb). Inbreeding times corresponding to each size class were estimated assuming a generation time of 3 years and a recombination rate of 1.5 cM/Mb (Thompson 2013; Kardos et al. 2018). The *x* axis is given in log scale. C) Proportion of ROH segments >500 kb (*F*_ROH_) in each individual’s genome.

In order to measure inbreeding levels across these ten sheep lineages, we studied ROH, which are continuous homozygous regions within an individual's genome derived from a recent common ancestor (Ceballos et al. 2018). We searched for segments of size >500 kb detected in individual genomes using PLINK (Chang et al. 2015). CYM and ANM genomes were among those groups with a relatively high ROH load, in terms of both the total number and size of the called segments (Fig. 4A, supplementary table S8, Supplementary Material online). Between the two, CYM had a higher load, in line with its relatively depleted diversity. We next studied the relative frequencies of four different ROH size classes, 0.5 to 1, 1 to 2, 2 to 3, and 3 to 5 Mb, which we used to estimate the time to the most recent common ancestor of each ROH class given the recombination rate and generation time estimates (Thompson 2013; Kardos et al. 2018). We had four time frames spanning from 200 to 20 years ago, assuming a generation time of 3 years (Fig. 4B). ANM and CYM had a mean ROH length of 0.63 and 0.82 Mb, which would be compatible with a common ancestor of these ROHs 53 and 41 generations ago (121 and 157 years ago), respectively. Neither carried segments >3 Mb and had relatively low proportions of segments of second and third class; this result suggests bottlenecks and small historical population size as sources of ROH rather than recent inbreeding. We also calculated the proportion of the genomes harboring ROH segments, referred to as *F*_ROH_ (Fig. 4C). Fourteen per cent of the ANM genome contained ROH segments, while CYM had a higher *F*_ROH_ of 20%, preceded by the EUM, which had the highest mean estimate of 42% among the sheep populations tested.

### Mutation Load

We further tested possible elevations in mutation load due to historic bottlenecks and small population sizes in CYM, ANM, and the other eight sheep lineages. For this, we assessed the substitutions in evolutionary conserved genomic regions, utilizing genomic evolutionary rate profiling (GERP) scores (Cooper et al. 2005). We chose stretches of sites with GERP scores > 4 as highly conserved regions. The relative mutation load (RML) for each sample was estimated by calculating the normalized GERP scores for the derived alleles observed in the conserved regions (von Seth et al. 2021).

**Fig. 5.**
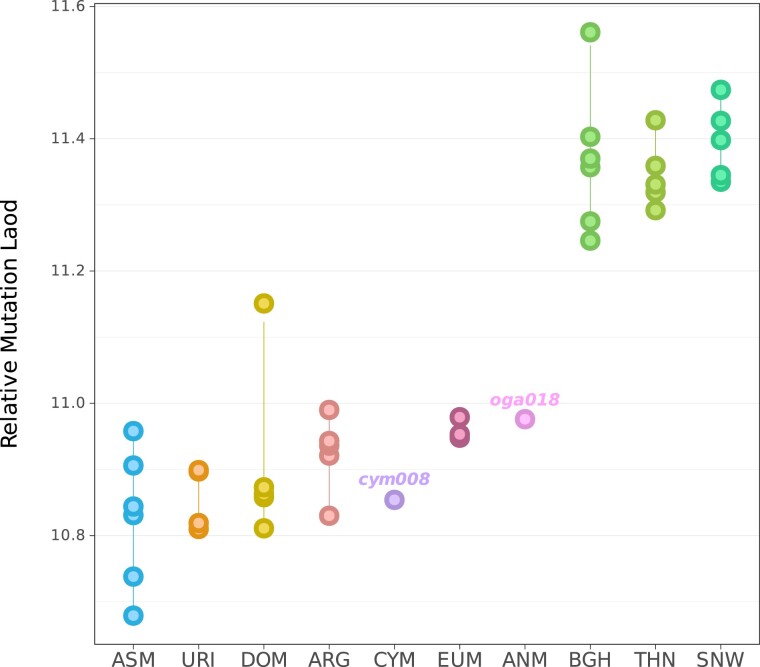
Mutation load estimates using GERP scores. RMLs were calculated as average GERP scores weighted by the number of derived variants, only including variants found in conserved regions (GERP > 4) (von Seth et al. 2021). We used goat alleles to infer the derived state. Only the high-coverage individuals oga018 and cym008 from the Anatolian and Cyprian populations, respectively, were included.

The load estimates showed substantial variation among individuals from the same taxon. Nevertheless, we did observe systematic patterns, with the lowest average load estimates among the ASM from Iran and the wild sheep from N. America and Siberia harboring the highest values (Fig. 5). Interestingly, CYM and ANM genomes had low-to-moderate RML estimates, with a slightly lower estimate for CYM. The discrepancies between degrees of diversity and mutation load may stem from differences in the duration and timing of the population bottlenecks (see Discussion).

### Coevaluation of Viability Metrics and Conservation Status

**Fig. 6.**
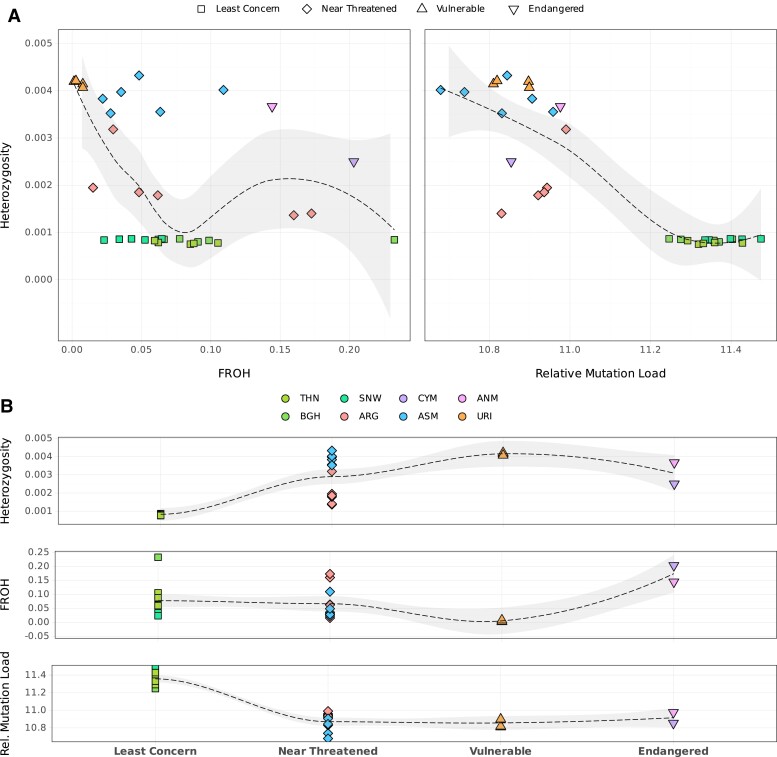
Coevaluation of genetic viability metrics and IUCN status. A) Correlations between viability metrics per individual compared across sheep lineages assessed by the IUCN. Regression lines were calculated using the method “loess” in the R stats package. B) The IUCN status compared with the genetic viability metrics heterozygosity (π), *F*_ROH_, and RML.

Finally, we assessed the conservation status of each population in relation to their studied viability metrics. DOM and EUM were not included since these two are not evaluated by the IUCN. First, we compared the individual heterozygosity estimates with the *F*_ROH_ values (Fig. 6A). We found moderate correlation between the two metrics (Spearman's *r* = −0.47, *P* = 0.007; Pearson's *r* = 0.29, *P* = 0.112). Meanwhile, the relationship between the RML and heterozygosity was strongly negative (Spearman's ρ = −0.79, *P* = 8.6e^−07^; Pearson's *r* = 0.89, *P* = 1.1e^−10^). Second, we compared the relationship between the IUCN status and the two viability estimates (Fig. 6B). In line with previous observations (Díez-del-Molino et al. 2018; Schmidt et al. 2023), the IUCN status was unrelated to genetic diversity levels among sheep lineages, with populations considered “Least Concern”, i.e. those from N. America/Siberia, having lower heterozygosity than the other groups labeled “Vulnerable”, “Near Threatened”, or “Endangered”. Intriguingly, the “Least Concern” populations also showed the highest mutation load estimates. Finally, we did not observe a strong relationship between IUCN status and *F*_ROH_, except that the “Endangered” ANM and CYM show the highest *F*_ROH_ values. Various interplays between demographic events such as introgression, founder effects, and isolation might explain this lack of relationship between the metrics (see Discussion).

## Discussion

### Phylogenetic Relationships between Anatolian and Cyprian Mouflons and DOM

Previous work reported that the ASM from Iran was the wild sheep lineage genetically closest to DOM, relative to other wild sheep, implying that ASM could have been the wild source of DOM (Hiendleder et al. 2002; Chessa et al. 2009; Cheng et al. 2023; Wang et al. 2023). Here, we report that ANM and CYM cluster with EUM and DOM, with ASM as outgroup, a pattern supported by MDS and *f_3_* and *f_4_* statistics. Further, we observed a higher affinity of DOM to CYM than to ANM. These results could be compatible with multiple scenarios: (i) the ancestors of ASM and ANM contributed equally to DOM, but recent URI introgression into ASM (Chen et al. 2021) differentiated ASM from the ANM-CYM-DOM cluster. (ii) The source of DOM was the ancestors of ANM and CYM but not ASM, and therefore, ANM and CYM share closer ancestry with EUM and DOM than ASM. This latter scenario would also be compatible with the suggestion that CYM could have been a proto-domesticate lineage. The positive *f_4_*(Goat, DOM; ANM, CYM) result is also consistent with the notion that CYM was an early feral lineage that split from the domesticated sheep gene pool. (iii) Recent DOM introgression into ANM and CYM is also possible given the significant positive *f_4_* statistics of the form *f_4_*(Goat, ANM; CYM, DOM) and *f_4_*(Goat, CYM; ANM, DOM). We are currently unable to reject any of these scenarios while noting that sheep ancient genomes (Yurtman et al. 2021) may be helpful for resolving this question.

### Differences in Demographic History between Anatolian and Cyprian Mouflons

Our data also allowed us to investigate the genomic footprints of the population size fluctuations via different viability metrics. Within-population diversity and individual-level heterozygosity estimates revealed CYM as harboring low-to-moderate and ANM as harboring moderate-to-high diversity values relative to other sheep lineages. It is intriguing that even though both subspecies ANM and CYM experienced bottlenecks of similar extent during similar time periods (Hadjisterkotis 2001; Özdirek 2009; Özüt 2009; Nicolaou et al. 2016; Michel and Ghoddousi 2020), their diversity levels are visibly different. The specifics of the bottleneck, such as its duration and how much of the original population structure survived, the extent of postbottleneck population growth, and the subsequent conservation practices may have affected these diversity-level differences. In addition, both populations have a history of population fluctuations due to paratuberculosis in ANM and keratoconjunctivitis and particularly poaching in CYM (Hadjisterkotis and van Haaften 1997; Toumazos and Hadjisterkotis 1997; Hadjisterkotis 2001; Hadjisterkotis 2002; Özdirek 2009; Özüt 2009; Michel and Ghoddousi 2020). Other than these more recent events, PSMC analyses suggest that shifts in the effective population sizes of ANM and CYM prior to 10 kya also follow different trajectories. To summarize, different processes might have shaped these diversity estimates, such as (i) CYM losing ancestral diversity due to founder effects during its transport to Cyprus or (ii) CYM undergoing serial bottlenecks in Cyprus, being isolated on an island. Both scenarios involve long periods of high homozygosity, which may have led to purging of recessive deleterious variants in CYM (Mathur et al. 2023; Robinson et al. 2023) and its consequent low RML compared with ANM.

We find the highest levels of ROH load in two CYM and ANM genomes relative to all other sheep lineages, except for EUM. Moreover, higher ROH in CYM than in ANM appears in agreement with the above scenarios involving smaller ancestral population size in CYM. Here, we note that our ROH analyses involve only one genome for ANM and CYM each. In both genomes, the majority of the segments is of moderate length, suggesting that recent inbreeding (mating between close relatives) is not the source of the high ROH load. Instead, the signal likely results from smaller effective population size (Kardos et al. 2017).

Beyond CYM and ANM, the sheep lineages studied here generally exhibit lower *F*_ROH_ than their distant wild cousins from the subfamilies Caprinae and Antilopinae (supplementary table S9, Supplementary Material online). Specifically, our *F*_ROH>1Mb_ estimates were 0.05% to 18% (median 3%), while species within Caprinae, such as musk-ox, were reported to harbor *F*_ROH>1Mb_ 25% to 75%, goats exhibit *F*_ROH>1Mb_ 10% to 25%, and the ibex was reported to have *F*_ROH>5Mb_ up to 10% (Grossen et al. 2017; Bertolini et al. 2018; Pečnerová et al. 2023). Within Antilopinae, populations of gazelle and oryx were found to show estimates of *F*_ROH>1.5Mb_ 20 to 50% and *F*_ROH>0.5Mb_ 10% to 50%, respectively (Alvarez-Estape et al. 2022; Humble et al. 2023). The lower levels of *F*_ROH_ in Ovis compared with its relatives may be attributed to population size and mating behavior differences, as well as to a possibly higher frequency of introgression events among different sheep lineages (Chen et al. 2021).

### Variable Diversity and Mutation Load Patterns among Wild and Domestic Sheep Genomes

Studying genetic viability metrics among all sheep lineages, we found that ASM and URI had the highest heterozygosity/diversity estimates, the lowest mutation loads, and on average the shortest ROH segments. These patterns may be consistent with the history of introgression between ASM and URI and/or domestic introgression to ASM (Carpenter et al. 2013; Morell Miranda et al. 2023).

EUM had exceptionally high *F*_ROH_ among the studied genomes. However, this result should be taken with caution as the genomes were sampled from a population in Finland transported from Sardinia/Corsica (Linnell and Zachos 2011); they may have thus undergone additional founder effects in the process. Meanwhile, the six DOM genomes had genetic characteristics similar to each other, with relatively high heterozygosity, low *F*_ROH_, and low mutation load (except for Awassi sheep which deviated from the rest with its high *F*_ROH_ likely due to management history).

The N. America/Siberia group (BGH, THN, SNW) showed systematically lower heterozygosity/diversity among all studied sheep lineages, harbored by far the highest mutation load estimates, and carried intermediate levels of ROH. These three lineages also had relatively small past *N*_e_ estimates in PSMC analyses, along with CYM and ARG. It is not clear why the N. America/Siberia group and CYM show disparate patterns with respect to mutation load, despite all three lineages being estimated to have long-term small *N*_e_.

Still, we note that our results should be taken with caution since the reference genome assembly represents a DOM genome, and therefore, heterozygosity/diversity estimates might be inflated/deflated depending on the populations' genetic proximities to DOM. In wild populations with higher proximity to DOM (e.g. CYM), more diversity may be represented, while in more distant populations (e.g. BGH), the estimates can be deflated. Mutation load estimates might also be affected by the asymmetric distances to the reference genome. Future work using graph genome or masked genome alignments (Koptekin, Yapar, et al. 2023; Koptekin, Yüncü, et al. 2023) may help address such possible biases.

### Comparison of Viability Metrics and Conservation Status

Our results reveal limited consistency between different genetic viability metrics among the studied sheep lineages. In contrast to expectation, we did not find a strong correlation between heterozygosity and the proportion of ROH segments. Recent inbreeding and introgression can be counted among the likely causes for the observed moderate relationship. The time passed since inbreeding might be too short to impact genome-wide diversities significantly. Admixture coupled with recent inbreeding might also elevate heterozygosity while also creating a high ROH load.

Regarding mutation load, we find a strong negative correlation with heterozygosity (Fig. 6A). Still, deviations from the general trend can be observed, such as the CYM with both low heterozygosity and low mutation load. Such discrepancies may emerge depending on the nature of bottlenecks and postbottleneck population growth, as well as the nature of mutation load, such as recessiveness and degree of deleteriousness. While long-term small population sizes may induce purging of recessive load, sudden bottlenecks can lead to the accumulation of deleterious mutations due to weakening selection. Here, too, possible impacts of introgression causing discrepancies cannot be excluded, as introgression may not only reduce but also contribute to the load if the introgressing population has a load of its own (Bertorelle et al. 2022).

We also observe differences in the chrX versus autosomal diversities between populations, indicating lower male versus female *N*_e_ among the ten sheep lineages, albeit at varying levels. Lower male *N*_e_ can be caused by male reproductive skew, which is most strongly observed for wild sheep from N. America/Siberia. Female philopatry and male dispersal are often observed in wild sheep populations (Garel et al. 2022), which may also contribute to sex differences in reproductive success and lead to low male *N*_e_. Meanwhile, the extent of this spatial behavior can depend on the habitat structure shaped by natural and anthropogenic factors, which may cause the observed differences among lineages. Sex-biased introgression from DOM and higher mortality of males due to natural causes or hunting pressure can also be counted among factors shaping chrX versus autosome diversity ratios.

Interestingly, we find a lack of a clear relationship between each lineage's IUCN status and their genetic diversity, ROH, and mutation load estimates. Since the assessment of IUCN status is based on current/recent population viability criteria and the genomic diversity indices mostly reflect historical population characteristics, a direct relationship may not be expected, especially if the population has undergone major demographic changes (Hare et al. 2011). Similar to recent work on a wider range of taxa, we did not find the genetic viability metrics to be indicative of the threat status among the eight sheep lineages evaluated by IUCN (Díez-del-Molino et al. 2018; Schmidt et al. 2023). Strikingly, although the N. America/Siberia group shows on average lower diversity levels and a distinctly high mutation load relative to other lineages, they are listed as “Least Concern”. Species from this group have relatively high census population size estimates (IUCN 2022), but our results suggest the possible vulnerability of these populations to perturbation, such as epidemics.

Finally, we touch upon the fact that EUM are currently not assessed by IUCN, since they are considered feralized descendants of DOM. Conservation of feralized species has been controversial, as in the case of the Australian dingoes. These were originally considered “vulnerable” but later discarded from the IUCN Red List as their status was revised as feral dogs, although conservation efforts for protecting some dingo populations are still ongoing (Elledge et al. 2006; Cairns et al. 2017; Jhala et al. 2018; Cairns 2021). Similarly, in the case of EUM, there have been local efforts to protect populations in Sardinia and Corsica (Mereu et al. 2019; Satta et al. 2021; Barbato et al. 2022; Portanier et al. 2022). These efforts are relevant given that the EUM has been in the wild for possibly 10k years, even if it may have experienced further domestic introgression after feralization. Harboring the largest amount of ROH segments among all studied sheep lineages, showing low diversity and high mutation load values, the EUM population (at least those individuals from Finland included in this study) seems to have a viability estimate lower than the officially endangered CYM and ANM. Considering the substantial domestic proximity of the CYM and ANM, we suggest a reassessment of the EUM’s conservation status.

## Materials and Methods

### Ethics Statement

This study did not include live animals. CYM tissue samples were collected only from animals found dead in the wild at the northern part of Paphos forest, located in the Troodos Mountains, mainly near the villages Kampos and Tsakistra, under the permit of the Ministry of the Interior for Scientific Research of the Republic of Cyprus.

### DNA Extraction, Library Preparation, and Sequencing

DNA extraction from tissue samples was performed using MACHEREY-NAGEL “NucleoSpin Tissue” kit following the standard protocol. DNA fragmentation via sonication was carried out using Qsonica Q800R at 100% amplitude for 15  On and 15  Off at 4 °C for 12 min. Fragmented DNA samples were quantified on Agilent Bioanalyzer 2100 to confirm an average 300 bp fragment length. If samples had an average fragment length longer than 300 bp, the sonication step was repeated. Dilutions were performed accordingly. Double-indexed Illumina sequencing libraries were prepared following the Meyer and Kircher protocol (Meyer and Kircher 2010) and sequenced on NovaSeq 6000 flowcells (NovaSeq Control Software 1.7.5/RTA v3.4.4) with a 101nt(Read1)-7nt(Index1)-7nt(Index2)-101nt(Read2) setup using the “NovaSeqXp” workflow in “S4” mode flowcell.

### DNA Data Processing and Variant Calling

Residual adapter sequences were removed using *AdapterRemoval v.2.3.1* (Schubert et al. 2016). Sequence reads were mapped to the sheep reference genome Oar_v4.0, using *BWA mem v.0.7.15* with the parameter *-M* (Li and Durbin 2010). After removing the PCR duplicates with *Picard MarkDuplicates*, reads with mapping qualities lower than 20 were discarded using *samtools v.1.9* (Li et al. 2009).

We chose one representative individual from each sheep population (Table 1), *n* = 10 in total, and downsampled them to similar coverages between 7.5 and 8.5× using *samtools v.1.9* (Li et al. 2009) *view -s*. We carried out de novo SNP calling using *GATK Haplotypecaller v.4.4.0.0* (Poplin et al. 2018), retaining SNPs with depths between 4× and 16× and a quality of 20, also using *-- maf 0.05* and *-- hwe 0.001*, followed by the genotyping of the remaining genomes (Table 1, supplementary table S1, Supplementary Material online). We included only biallelic SNPs and did filtering using *bcftools v.1.18* (Li 2011) with parameters *QUAL>=20 QD>=2.0 SOR<=3.0 FS<=60.0 MQ>=40.0 MQRankSum>-12.5 ReadPosRankSum>-8.0*. After the filtering steps, the resulting autosomal data set contained a total of 14,237,712 SNPs and the chrX data set contained 427,454 SNPs. We used these sets of SNPs in the calculation of *f*-statistics, identification of autosomal ROH segments, and the genetic relatedness analysis with *READ*. We also validated our main findings based on *f_3_* statistics using a subset of our autosomal data set. For this, we only included the sites that were heterozygous in the outgroup goat genome (Table 1), which resulted in a total of 116,613 SNPs.

### Sex Determination

We determined the genetic sex of the studied genomes utilizing the *R_x_* metric (Mittnik et al. 2016). This is the ratio of chrX coverage to mean coverage across autosomes. With a complete genome assembly, the ratio is expected to be 1 for females and 0.5 for males. We calculated the *R_x_* value for each genome in our data set. Studying the data revealed a bimodal distribution, as expected (supplementary fig. S10, Supplementary Material online). We then used *k-means* unsupervised clustering to define *R_x_* clusters with the *k-means* function in R (R Core Team 2021). We observed that the mean *R_x_* values for the two cluster for females and males were 0.74 and 0.42, respectively (supplementary fig. S10, Supplementary Material online). The deviation from the expected 1 and 0.5 values is likely due to genome assembly issues. Based on this result, we redefined thresholds for female and male assignment as 0.65 and 0.5, respectively, following the general approach of Mittnik and colleagues for human data (Mittnik et al. 2016). More specifically, we assign genomes with *R_x_* − 1.96SE > 0.65 as XX and those with *R_x_* + 1.96SE < 0.5 as XY, where SE stands for standard error calculated using variance among autosomes (Mittnik et al. 2016). Our script for sex determination using *R_x_* and these thresholds is available at https://github.com/mskilic/SexDetermineOar.

### Relatedness Estimation

For the relatedness estimates, we used the program *READ* which detects up to second-degree relatives using pseudo-haploid genotype data (Kuhn et al. 2018). The pairwise mismatch (P_0_) values were normalized using the median of the P_0_ estimates of each population. Then, normalized P_0_ values were subtracted from 1 to obtain θ (kinship coefficient) estimates. We assigned the individual pairs to their respective kinship degrees using the midpoint between the two expected θ values (θ_1_ and θ_2_) as a threshold, calculated as (θ_1_ + θ_2_)/2 (Kuhn et al. 2018)) (supplementary table S3, Supplementary Material online). One of the identical genomes (cym012) and the potential second- and third-degree relatives that appeared divergent in the pairwise *f_3_* estimates (cym006 and cym007) were excluded from the diversity analyses.

### mtDNA Analyses

mtDNA gVCF files were generated using *bcftools v.1.18* (Li 2011) with parameters *-q20 -Q20 -mV indels*. Average mitochondrial pairwise differences (π) within each population were calculated using the formula:


$$\pi \;=\;\frac{1}{[n(n-1)]\;/\;2}\times \frac{\sum_{i<j}\;\pi_{ij}}{L}\;,$$

where *n* is the total number of individuals in each population, *L* is the total number of sites, and *π_ij_* is the number of nucleotide differences for each pair. Only the biallelic sites which were nonmissing in at least two individuals within the population were taken into account.

Mitogenome consensus sequences were generated from BAM files using *ANGSD v.0940* (Korneliussen et al. 2014) with parameters “-doFasta 2”, “-minQ 30”, “-minMapQ 30”, and “-setMinDepth 2”. Ten representative DOM mitogenomes with known hpg (two for each hpg) with NCBI GenBank accession no.: HM236174-83 (Meadows et al. 2007) were also added to the data set. Consensus sequences were aligned with *MAFFT v.7.490* (Katoh and Standley 2013), and a MJ network (Bandelt et al. 1999) was constructed with *PopART* (Leigh and Bryant 2015).

### Outgroup-*f*_3_ and *f*_4_ Statistics

We calculated outgroup-*f_3_* and *f_4_* statistics using *Admixtools v.2.0.0* (Maier and Patterson 2023) with default parameters and *maxmiss = 1* (includes all SNPs). We used goat as the outgroup (Table 1). *f_3_* statistics of the form *f_3_*(Goat, ind1, ind2) was performed for the estimation of within-population (interindividual) genetic diversities. *f_3_*(Goat, pop1, pop2) was calculated for estimating population differentiation, pop1 and pop2 corresponding to different wild sheep populations. When comparing populations, we preferred outgroup-*f_3_* instead of F_ST_ because the latter is sensitive to population size fluctuations and consequent variation in within-population diversity, while the former is not (Patterson et al. 2012); *f_3_* can therefore capture population divergence and admixture more effectively than F_ST_ (Koptekin, Yapar, et al. 2023; Koptekin, Yüncü, et al. 2023).

To summarize the genetic relationships among populations, we used pairwise 1-outgroup-*f_3_* values to construct a distance matrix, which we used to perform MDS and also construct an NJ tree. MDS was run using the function *cmdscale* implemented in R package *stats*. For the NJ tree, the function *nj* in the R package *ape v.5.7-1* (Paradis and Schliep 2019) was utilized. We performed *n* = 500 bootstraps by first dividing the genotype data into chunks of 30,000 SNPs, randomly sampling chunks with replacement and calculating outgroup-*f_3_* statistics per iteration. We rooted the NJ tree by adding the outgroup goat to the 1-*f_3_* distance matrix manually, represented by a distance of 1 to all sheep lineages.

### Demographic History Reconstruction

We used the PSMC model (Li and Durbin 2011) to infer the changes in historical effective population sizes. PSMC was run for the highest coverage CYM and ANM individuals with parameters –N25 –t15 –r5 –p ‘4 + 25 * 2 + 4 + 6, using a generation time of 3 years, and a mutation rate of 1.51 × 10e^−8^ for scaling (Zhao et al. 2017; Chen et al. 2019; Lv et al. 2021). We also ran PSMC with the same genomes downsampled to similar coverages (7.5 to 8.5×).

### ROH and Heterozygosity Estimates

We used *ANGSD v.0.940* (Korneliussen et al. 2014) for the estimation of genome-wide heterozygosities, with parameters *-dosaf 1 -GL 1 -doCounts 1 -minmapq 20 -minq 20 -uniqueonly 1 -remove_bads 1*. All genomes were downsampled to similar coverages (6.5 to 7.5×) using *samtools v.1.9* (Li et al. 2009) *view -s*, prior to heterozygosity estimation. For the identification of ROH segments, we used *PLINK v.1.9* (Chang et al. 2015) with parameters “*--homozyg-window-snp 50 --homozyg-window-het 1 --homozyg-snp 30 --homozyg-kb 500 --homozyg-density 30*”, which represent a minimum ROH length of 0.5 Mb. We calculated *F*_ROH_, the proportion of the genome containing ROH segments, as the sum of ROH segments divided by the total size of the sheep reference genome. We grouped the ROHs into different size classes (supplementary fig. S11, Supplementary Material online). In order to estimate the time period of inbreeding corresponding to each size class, we used the formula *g = 100/2rL* (Thompson 2013; Kardos et al. 2018), where *g* corresponds to generation time, *r* to recombination rate, and *L* to ROH length in Mb. We also estimated inbreeding time using the mean ROH length in CYM and ANM genomes. We used 1.5 cM/Mb as the recombination rate and calculated the estimated times using a generation time of 3 years.

### Mutation Load and GERP Scores

We downloaded GERP scores from the Ensembl database, which were calculated for DOM reference Oar_v3.1 (Martin et al. 2023). Using the UCSC liftover tool, we mapped the conservation scores to positions on the reference Oar_v4 (Nassar et al. 2023). The ancestral states were determined based on the alleles observed in the goat genome. We calculated the mutation load for each derived allele in sheep genomes in conserved regions of the genome, following von Seth and colleagues (von Seth et al. 2021). For this, we first defined conserved genomic regions in the reference genome as strings of consecutive bases with GERP > 4. We then calculated the RML (von Seth et al. 2021) for each genome using:


$${\mathrm{RML}}_{x}=\;\frac{\sum_{i=1}^{k}\;n_{ix}\;\times \;g_{i}}{N_{x}},$$

where *x* is one sheep genome, *k* corresponds to the total number of conserved regions, *g* is the GERP score for each region *i*, *n* is the number of derived alleles in region *i* in genome *x*, and *N* corresponds to the total number of derived alleles in genome *x*. The number of derived alleles was counted as one for heterozygous sites and as two for homozygous sites.

## Supplementary Material

evae090_Supplementary_Data

## Data Availability

All newly generated sequence data were submitted to the European Nucleotide Archive (ENA) with the project ID PRJEB69690.
