# Supplementary material for: Population Genomic History of the Endangered Anatolian and Cyprian Mouflons in Relation to Worldwide Wild, Feral, and Domestic Sheep Lineages
Source: Genome Biol Evol. 2024 Apr 27;16(5):evae090. doi: 10.1093/gbe/evae090 (PMC11109821; doi:10.1093/gbe/evae090)
Supplement: evae090_Supplementary_Data [file evae090_supplementary_data.zip › mouflon_suppfigures_revision.docx]

**Supplementary Figures**


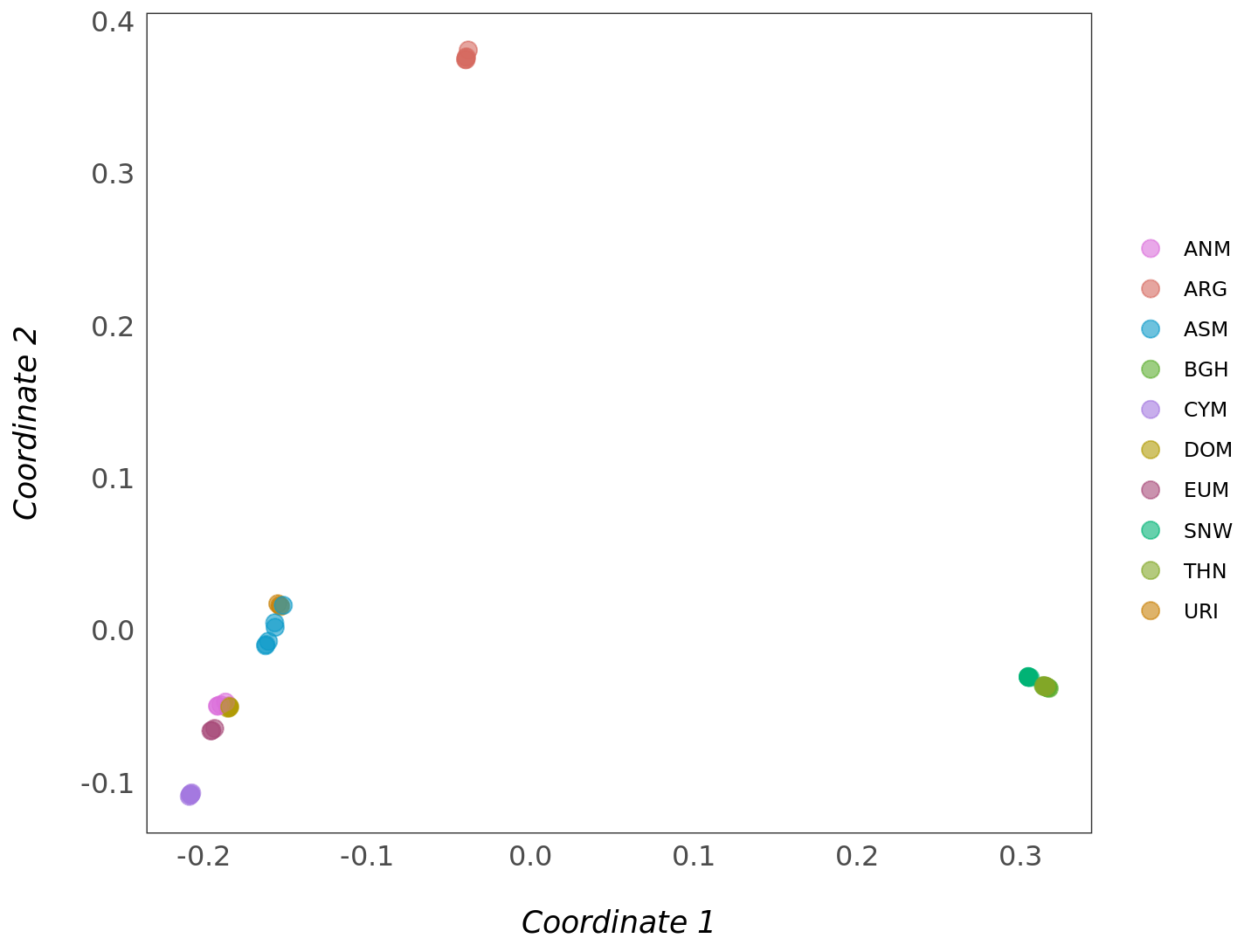


**Figure S1. MDS analysis of the studied individuals.** Multidimensional scaling using 1 - *outgroup-f_3_* values between individuals as distance proxies, with goat as the outgroup. Coordinate 1 versus 2 is plotted.


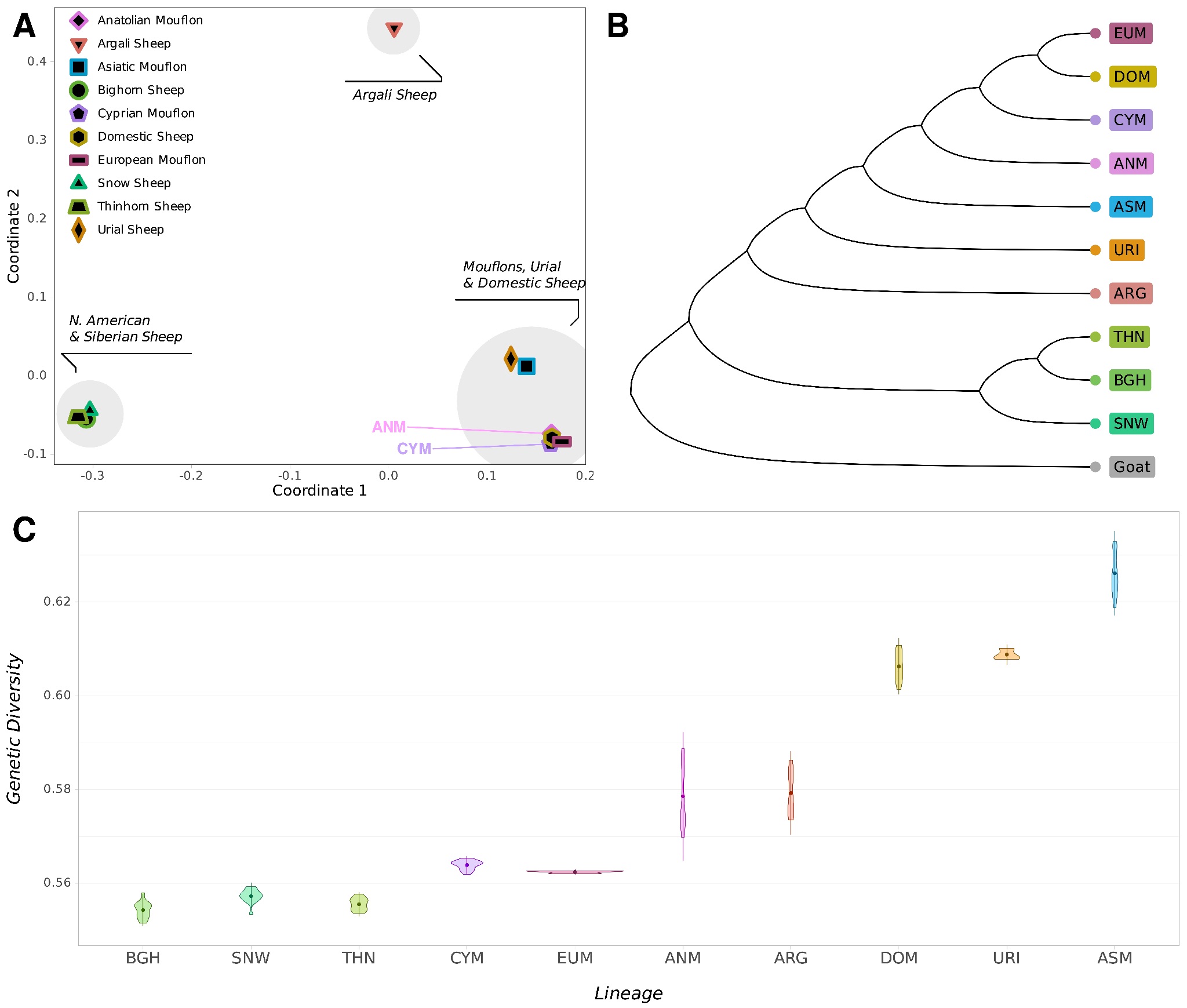


**Figure S2. Phylogenetic relationships between these sheep lineages and diversity estimates using the dataset of goat heterozygous SNPs.** (A) Multidimensional scaling (MDS) analysis of the studied sheep lineages, using 1- outgroup f_3_ statistics as distance proxies. (B) Neighbour-joining (NJ) tree of the studied sheep lineages, using (1 - outgroup f_3_) as distance proxies. (C) Within-population diversity values estimated using pairwise 1 - outgroup f3 statistics per lineage.


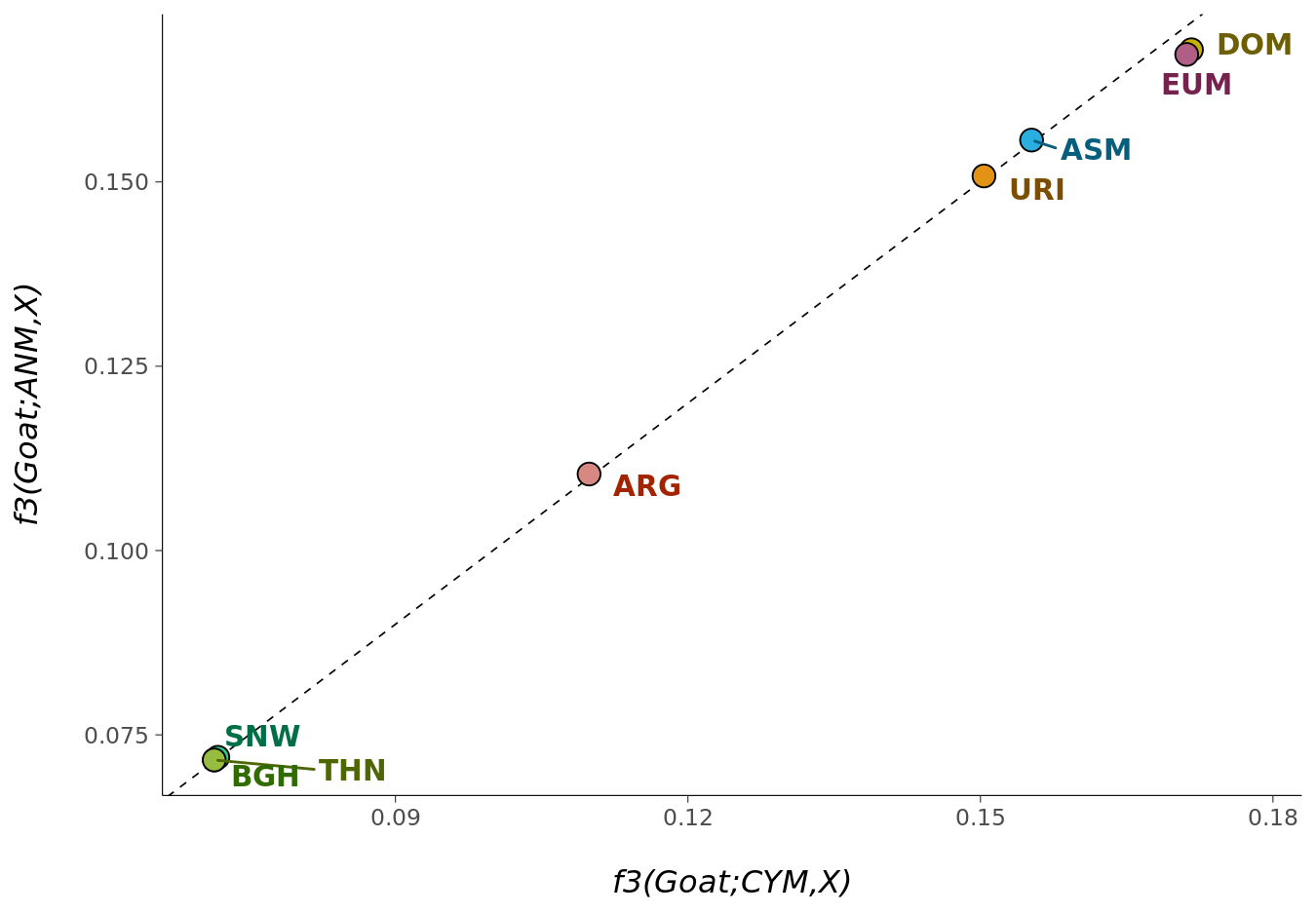


**Figure S3. Comparison of *f_3_* statistics between CYM/ANM and other sheep lineages.** Outgroup*-f_3_* statistics of the form *f_3_(Goat;CYM,X)* on the x-axis plotted against *f_3_(Goat;ANM,X)* on the y-axis where X corresponds to other studied sheep lineages. Points on the dotted line represent similar affinity of both CYM and ANM to the other lineages (Spearman’s ρ=1, p=5e-05).


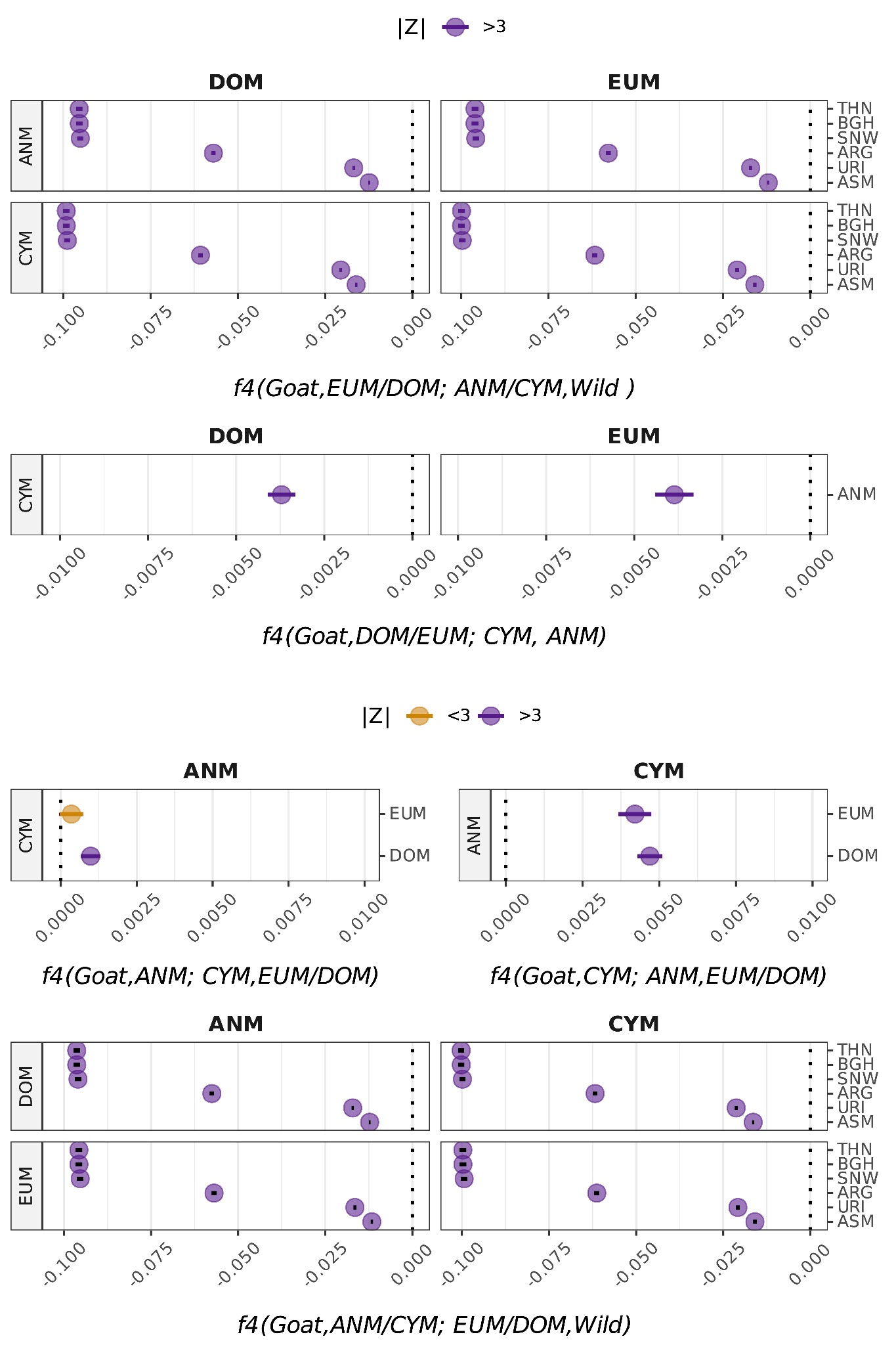


**Figure S4. *f_4_* statistics between CYM, ANM, DOM and EUM.** f4 statistics of the form *f_4_(Goat, EUM/DOM, ANM/CYM, Wild), f_4_(Goat, EUM/DOM; ANM,CYM), f_4_(Goat, ANM/CYM; ANM/CYM, EUM/DOM)* and *f_4_(Goat, ANM/CYM,; EUM/DOM,Wild)*, where Wild corresponds to other studied sheep lineages. Orange color depicts results with |Z| < 3, and purple depicts significant results with |Z| > 3.


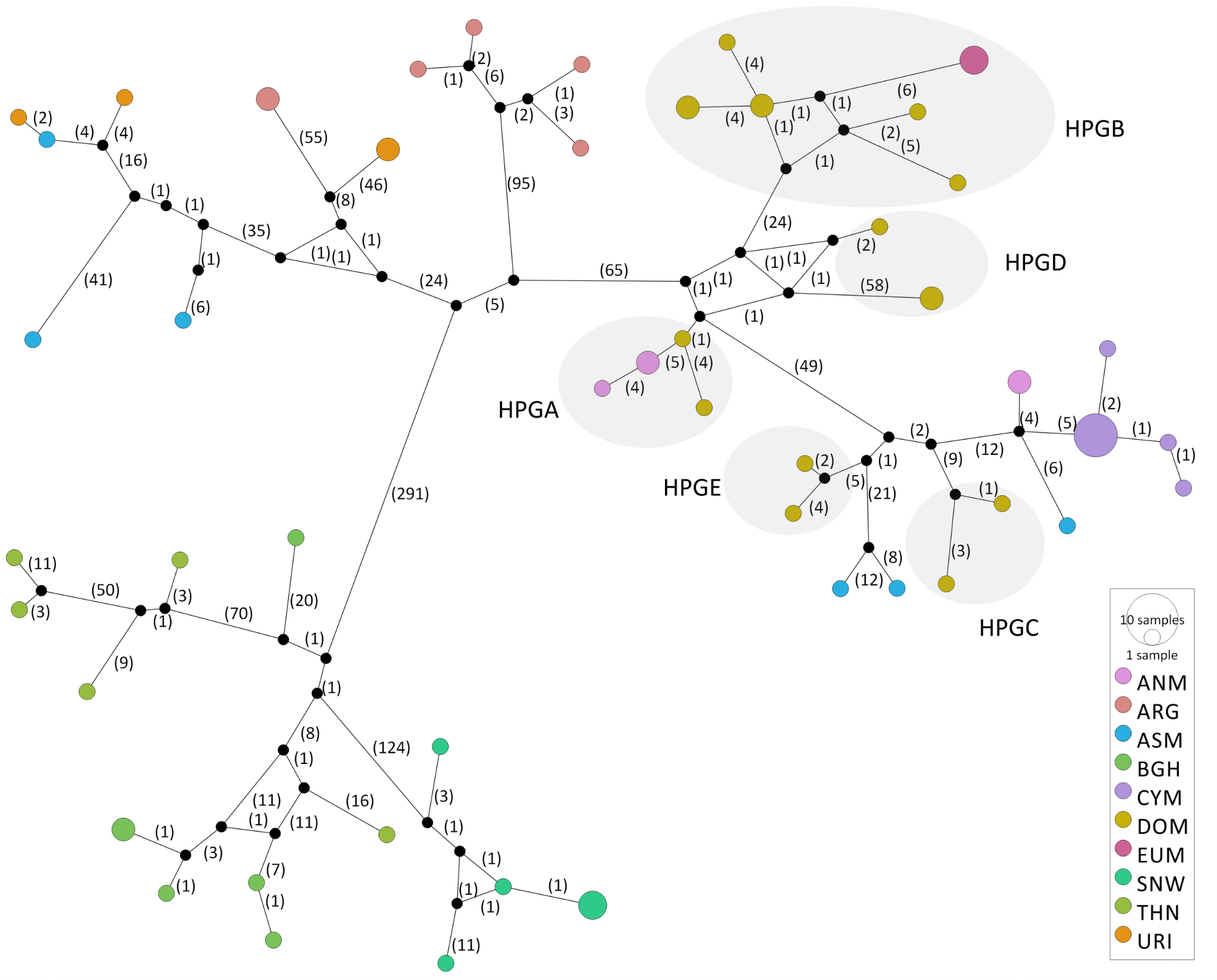


**Figure S5.** **Mitogenome DNA median-joining network (MJN) of wild and domestic sheep.** Node sizes are proportional to the number of samples in the node and numbers on edges show the number of nucleotide differences between nodes. Domestic haplogroup clusters were denoted as gray circles. Black dots represent hypothetical nodes.


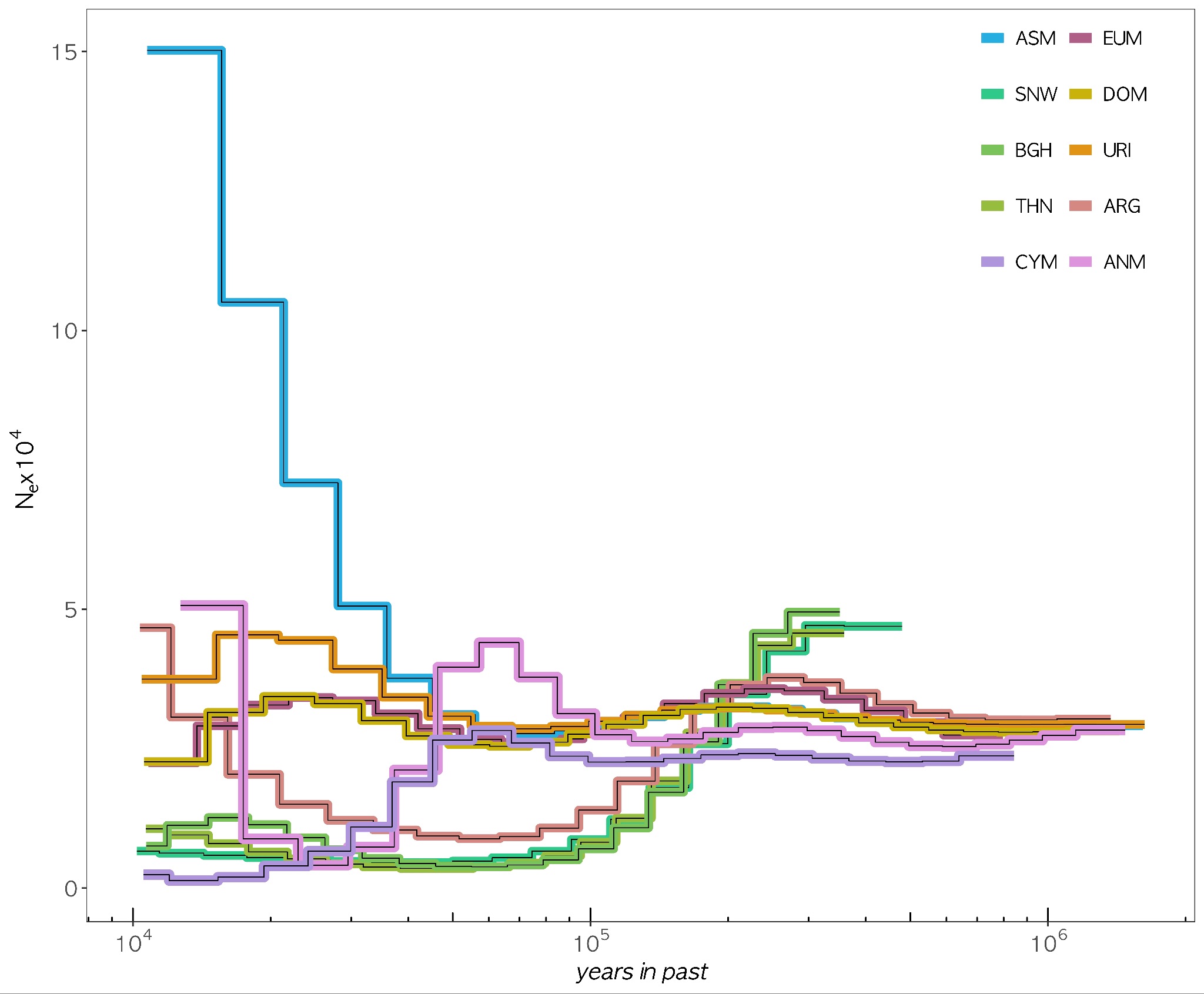


**Figure S6. PSMC analysis of genomes downsampled to similar coverages.** The genomes were downsampled to coverages 7.5-8.5x. Analysis was carried out assuming a generation time of 3 years and a mutation rate of 1.5x10^-8^. The x-axis shows time in a log scale, the y-axis shows the estimated effective population size.


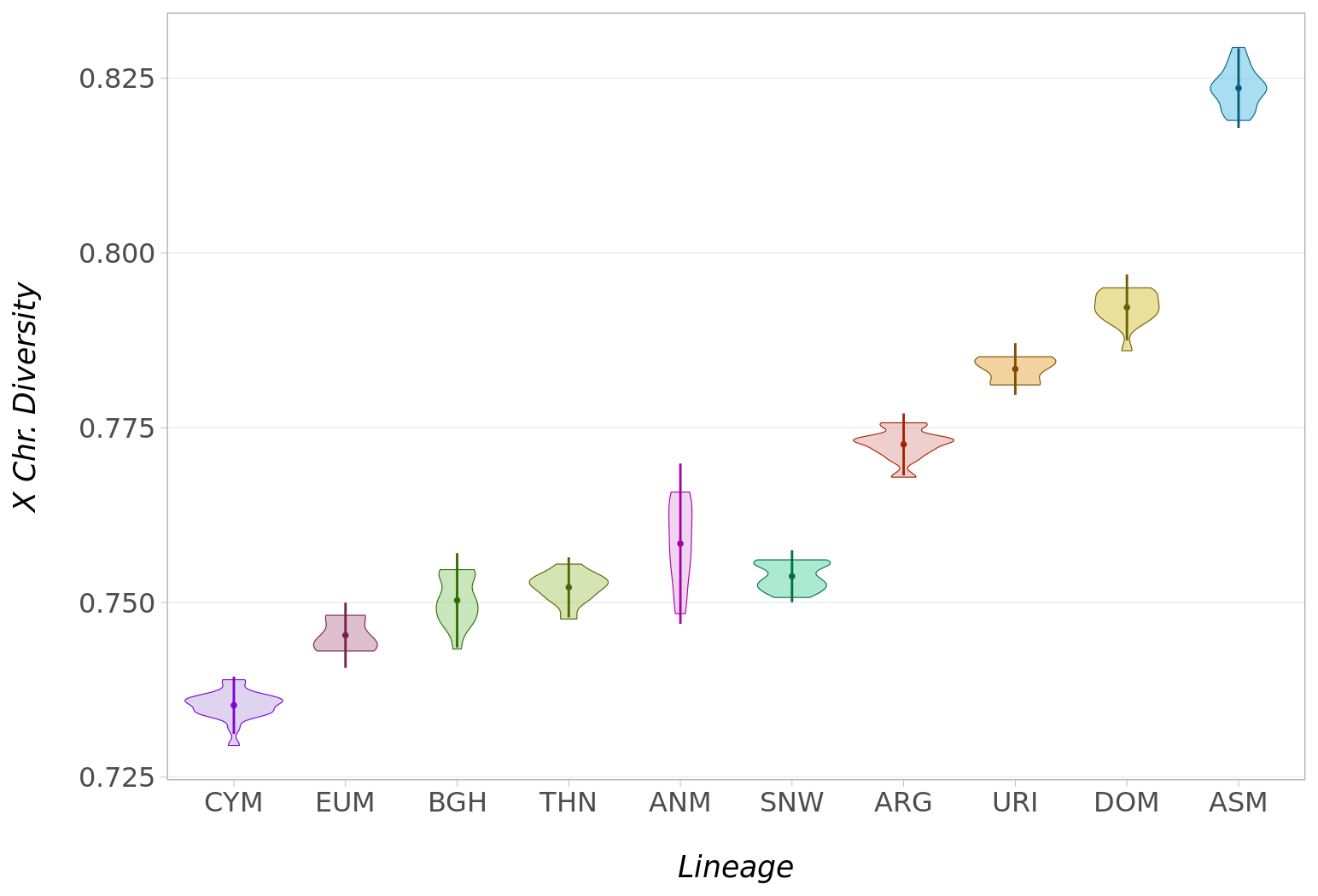


**Figure S7. X chromosomal diversities of the studied sheep lineages.** Within-population chrX diversity values estimated using pairwise 1 - outgroup-f3 statistics per lineage.


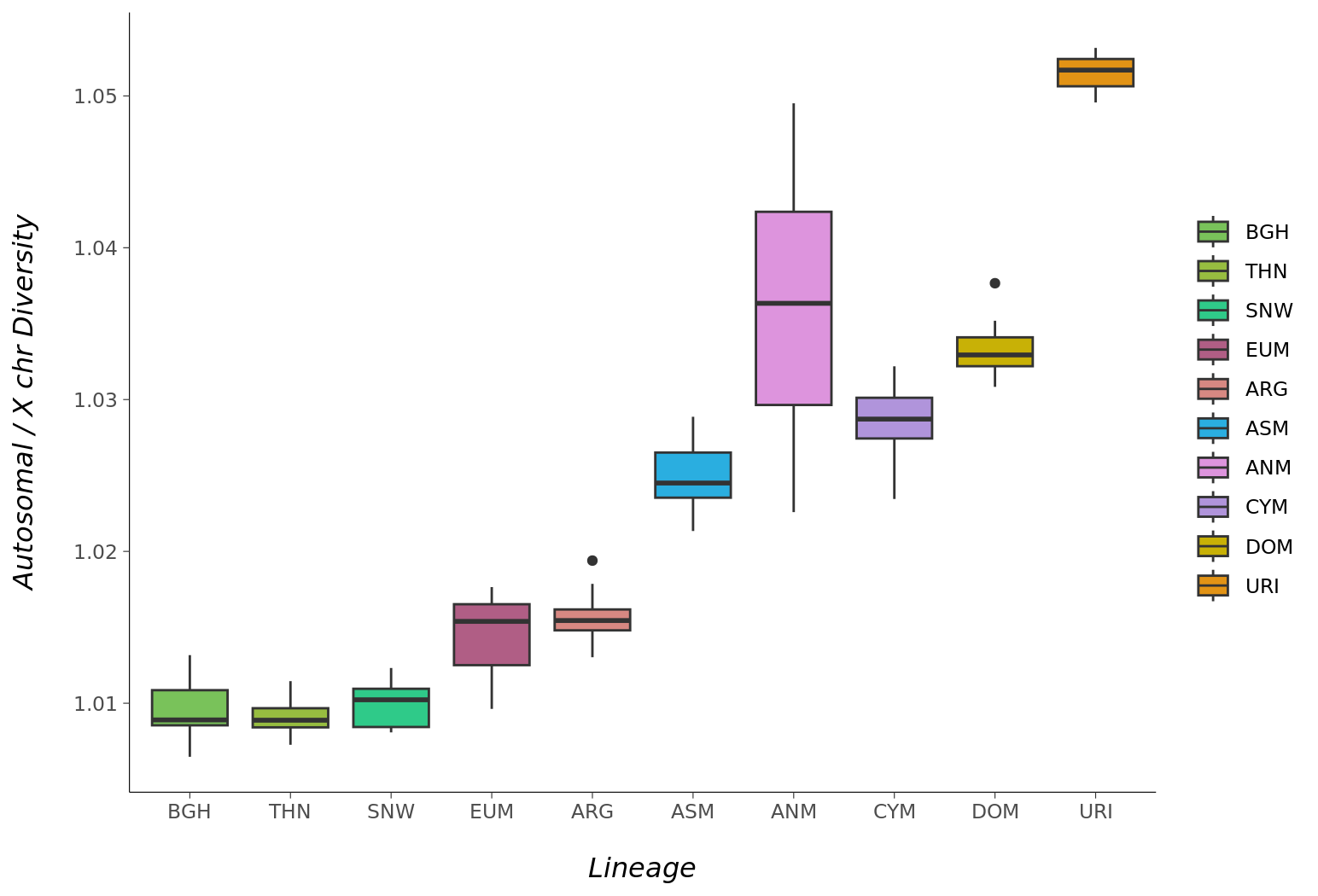


**Figure S8. Proportion of autosomal / chrX diversities of the studied sheep lineages.** Within-population autosomal and chrX diversity values estimated using pairwise 1 - outgroup-f3 statistics per lineage.


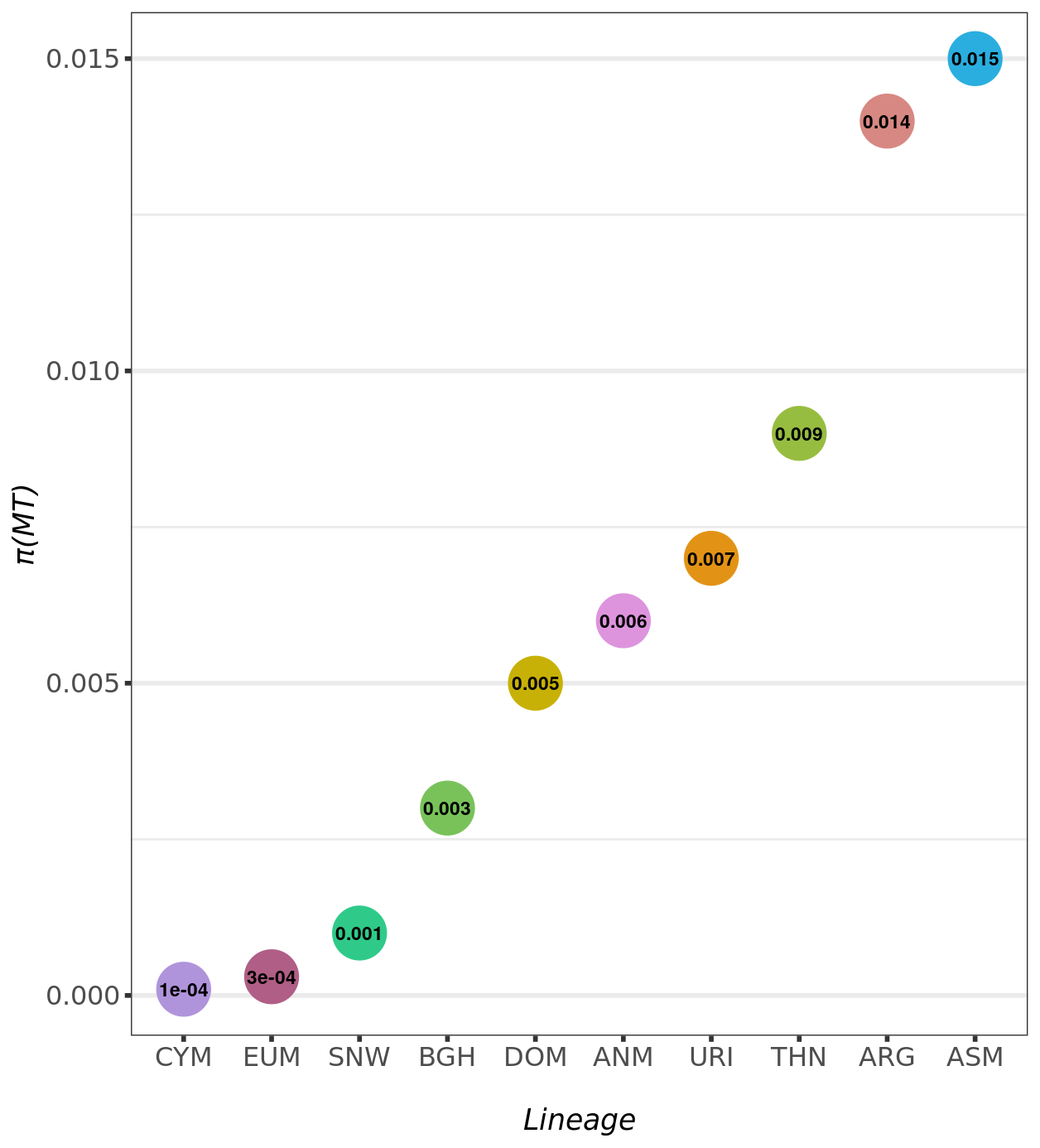


**Figure S9. Mitochondrial diversities of the studied sheep lineages.** Within population pi (π) estimates using mitochondrial genomes.


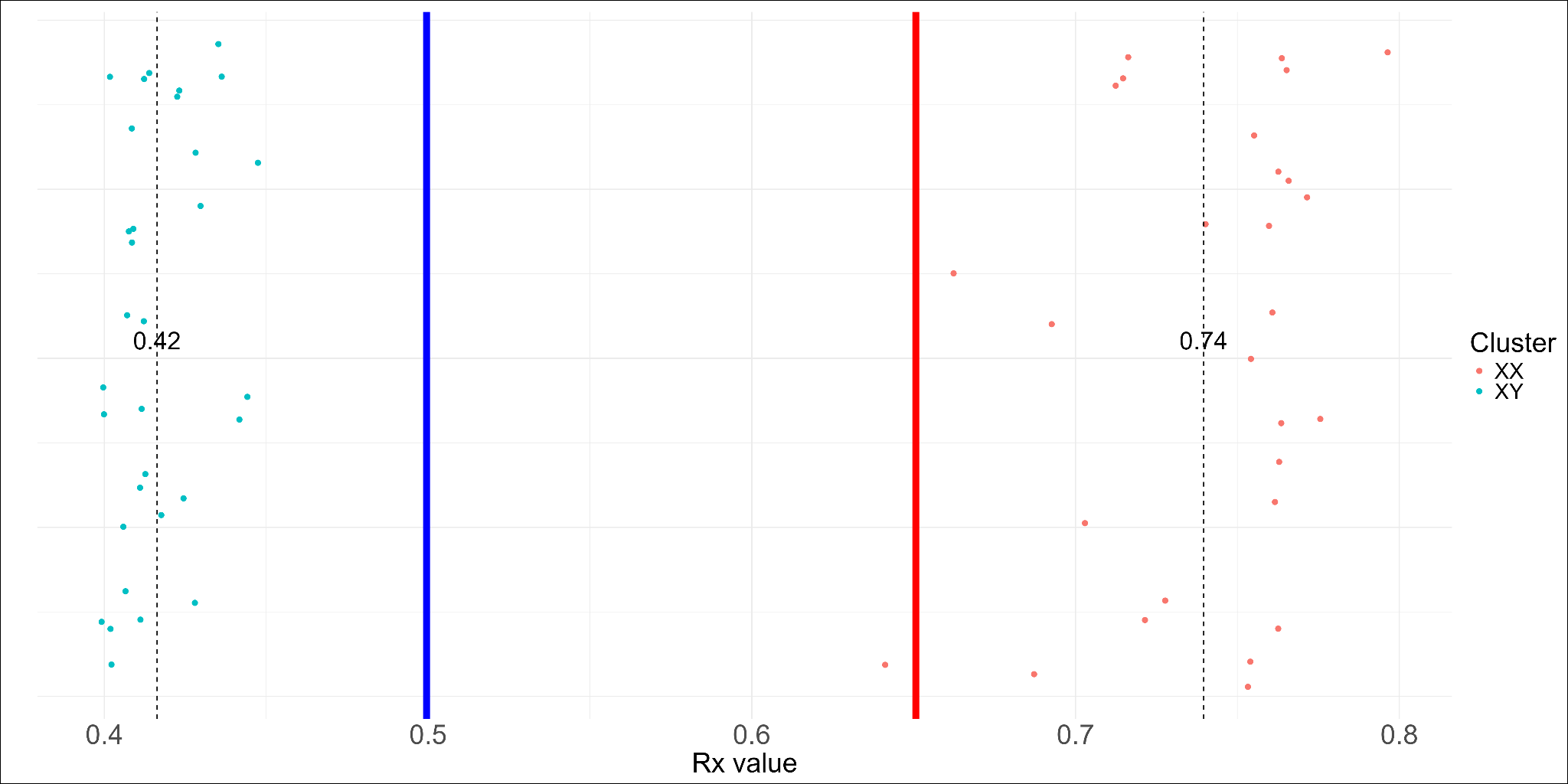


**Figure S10. K-means clustering of the *R_x_* values of the studied sheep lineages.** Dashed lines represent the means of the clusters. Colored bold lines indicate the thresholds we defined for our SexDetermineOar algorithm. Each point corresponds to the *R_x_* value of a sheep genome.

**
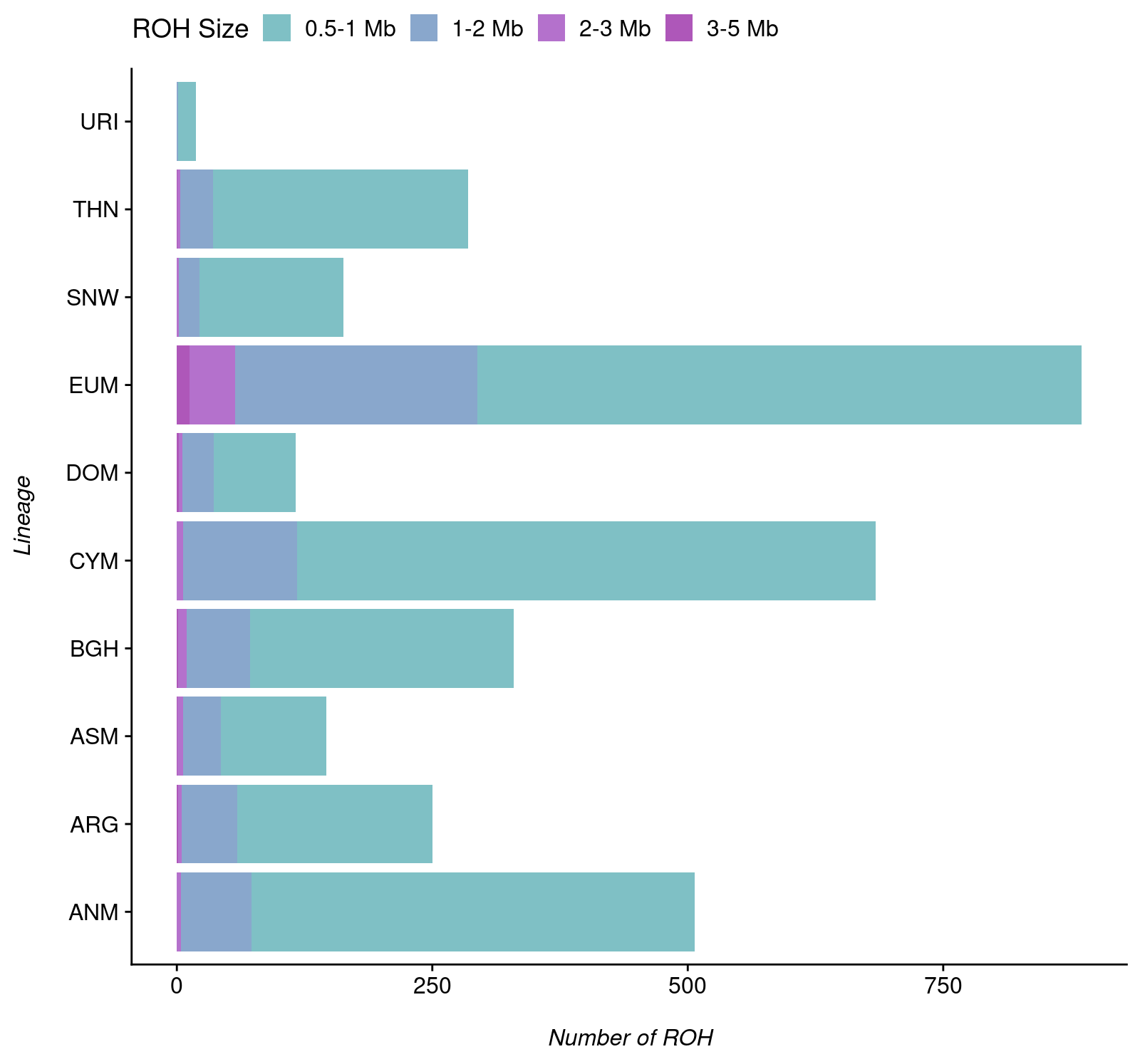
**

**Figure S11. ROH load per size category amongst the sheep lineages.**  Size distribution of ROH segments divided into four classes (0.5-1 Mb, 1-2 Mb, 2-3 Mb, 3-5 Mb). The x axis corresponds to the mean number of ROH segments of each class per lineage.
